# The interaction of β-arrestin1 with talin1 driven by endothelin A receptor as a feature of α5β1 integrin activation in high-grade serous ovarian cancer

**DOI:** 10.1038/s41419-023-05612-7

**Published:** 2023-01-30

**Authors:** Ilenia Masi, Flavia Ottavi, Danila Del Rio, Valentina Caprara, Cristina Vastarelli, Sara Maria Giannitelli, Giulia Fianco, Pamela Mozetic, Marianna Buttarelli, Gabriella Ferrandina, Giovanni Scambia, Daniela Gallo, Alberto Rainer, Anna Bagnato, Francesca Spadaro, Laura Rosanò

**Affiliations:** 1 Institute of Molecular Biology and Pathology, CNR, Rome, 00185 Italy; 2 Unit of Preclinical Models and New Therapeutic Agents, IRCCS—Regina Elena National Cancer Institute, Rome, 00144 Italy; 3 Department of Engineering, Università Campus Bio-Medico di Roma, via Álvaro del Portillo 21, Rome, 00128 Italy; 4 Institute of Nanotechnology (NANOTEC), National Research Council (CNR), c/o Campus Ecotekne, via Monteroni, Lecce, 73100 Italy; 5 San Raffaele Hospital, Division of Neuroscience, Institute of Experimental Neurology, San Raffaele Scientific Institute, Via Olgettina, 60, Milan, 20132 Italy; 6 Dipartimento Universitario Scienze della Vita e Sanità Pubblica-Sezione di Ginecologia ed Ostetricia—Università Cattolica del Sacro Cuore, Largo A. Gemelli 8, Rome, 00168 Italy; 7 Dipartimento Scienze della Salute della Donna, del Bambino e di Sanità Pubblica, Fondazione Policlinico Universitario A. Gemelli, IRCCS, Largo A. Gemelli 8, Rome, 00168 Italy; 8 Confocal Microscopy Unit, Core Facilities, Istituto Superiore di Sanità, Rome, 00161 Italy

**Keywords:** Ovarian cancer, Cancer, Cell biology

## Abstract

Dissemination of high-grade serous ovarian cancer (HG-SOC) in the omentum and intercalation into a mesothelial cell (MC) monolayer depends on functional α5β1 integrin (Intα5β1) activity. Although the binding of Intα5β1 to fibronectin drives these processes, other molecular mechanisms linked to integrin inside-out signaling might support metastatic dissemination. Here, we report a novel interactive signaling that contributes to Intα5β1 activation and accelerates tumor cells toward invasive disease, involving the protein β-arrestin1 (β-arr1) and the activation of the endothelin A receptor (ET_A_R) by endothelin-1 (ET-1). As demonstrated in primary HG-SOC cells and SOC cell lines, ET-1 increased Intβ1 and downstream FAK/paxillin activation. Mechanistically, β-arr1 directly interacts with talin1 and Intβ1, promoting talin1 phosphorylation and its recruitment to Intβ1, thus fueling integrin inside-out activation. In 3D spheroids and organotypic models mimicking the omentum, ET_A_R/β-arr1-driven Intα5β1 signaling promotes the survival of cell clusters, with mesothelium-intercalation capacity and invasive behavior. The treatment with the antagonist of ET_A_R, Ambrisentan (AMB), and of Intα5β1, ATN161, inhibits ET-1-driven Intα5β1 activity in vitro, and tumor cell adhesion and spreading to intraperitoneal organs and Intβ1 activity in vivo. As a prognostic factor, high EDNRA/ITGB1 expression correlates with poor HG-SOC clinical outcomes. These findings highlight a new role of ET_A_R/β-arr1 operating an inside-out integrin activation to modulate the metastatic process and suggest that in the new integrin-targeting programs might be considered that ET_A_R/β-arr1 regulates Intα5β1 functional pathway.

## Introduction

The development of cancer metastasis depends on the sites of interaction between cancer cells and their surrounding microenvironment, which serve as dynamic signaling hubs that regulate cellular adaptations during different steps [1]. Many of these interactions are established by integrins, the main extracellular matrix (ECM) receptors of cell adhesion, which provide vital cues by sampling the chemical and physical environmental conditions [2, 3]. As bidirectional signal transducers expressed in tumor/stromal cells, integrins are 24 heterodimers composed of a combination of 18 α- and 8 β-subunits. While the extracellular domains bind distinct ECM proteins, the cytoplasmic tails bind to the cytoskeleton and contain binding sites for multiple integrin-binding proteins required for its signaling and function [2, 3]. The binding of ligands to the extracellular domains leads to the formation of a large heterogeneous multiprotein signaling platform, the integrin adhesion complex, tethering integrins and ECM to the actin cytoskeleton through the recruitment of adapter proteins, talins, tensins, and kindlins, to the β cytoplasmic tail, transducing the complex outside-in signaling [3]. The prevailing view of integrin activation is called inside-out activation, whereby intracellular signals can be induced by talin that rapidly regulates integrin affinity for ligands, highlighting that the binding of talin is a common step for outside or inside integrin activation [3].

In this framework, G-proteins may mediate integrin signaling. Indeed, both integrin ligands and G protein-coupled receptors (GPCRs) activate G_α__13_ and promote binding to integrin β subunits, including β1 and β3, which in turn interact with intracellular molecules, including talin, kindlin, and c-Src [4, 5]. In hematopoietic and non-hematopoietic cells, GPCRs employ G-proteins to initiate “inside-out” signaling and cell adhesion [4–7]. However, the mechanism by which G_α13_ and other GPCR-linked signaling molecules bind to integrin β subunits remains unknown.

Intraperitoneal dissemination is the primary means of SOC metastasis, and the direct spread of tumor cells into the peritoneal cavity is due to enhanced anchorage-independent tumor cell survival, which may be supported by integrins [8]. In this context, elevated expression of integrin α5β1 (Intα5β1) correlates with increased metastatic potential and shortened high-grade (HG)-SOC patient survival [8–12], highlighting the primacy of the β1 subunit in this tumor. When establishing secondary tumors, single cells or spheroids must attach to the mesothelium through interactions with Intα5β1 and underlying ECM proteins. In the final metastatic step, tumor cells must penetrate the mesothelial surfaces and degrade the ECM within the basement membrane underlying the peritoneum, omentum, and abdominal organs through a process called mesothelial cell (MC) clearance [13]. Ovarian cancer spheroids use integrin- and talin-dependent activation of myosin traction force to promote MC clearance, highlighting the importance of integrin-mediated actomyosin contraction in overcoming cell–cell attachment and promoting ECM enzymatic degradation [14, 15].

In SOC, a member of the GPCR family, receptor A (ET_A_R) for endothelin-1 (ET-1) provides an active signaling network that controls pro-invasive and metastatic features, including cytoskeletal activity, changes in shape, and invasive protrusions [16–20]. Indeed, ET-1 derived from tumor and stromal cells activates receptor-mediated promigratory signaling, thereby inducing cancer cell invasion. The scaffold protein β-arrestin1 (β-arr1) integrates adhesion and proteolytic signaling through its interaction with the integrin-related protein, integrin-linked kinase (ILK), which allows cells to remodel the ECM and invade, and facilitate crosstalk with MCs [17]. The interaction between ILK and β-arr1 is a prerequisite for tumor cells to bypass the mesothelial barrier and invade through an invadopodia-mediated mechanism. However, the existence of a direct interaction between ET_A_R/β-arr1 and the upstream integrin is not understood. Considering the interplay between GPCR and integrin signaling and the ability of β-arr1 to interact with components of the adhesome, in this study, we aimed to define a possible β-arr1/ET_A_R-driven inside-out Intβ1 activation to support MC clearance and stromal invasion. We also investigated in vitro and in vivo targeting of ET_A_R with Ambrisentan and Intα5β1 with ATN161 to inhibit Intα5β1 and prevent the metastatic spread of SOC cells.

## Results

### High ITGB1/EDNRA expression correlates with the poor prognosis of HG-SOC patients

**Fig. 1:**
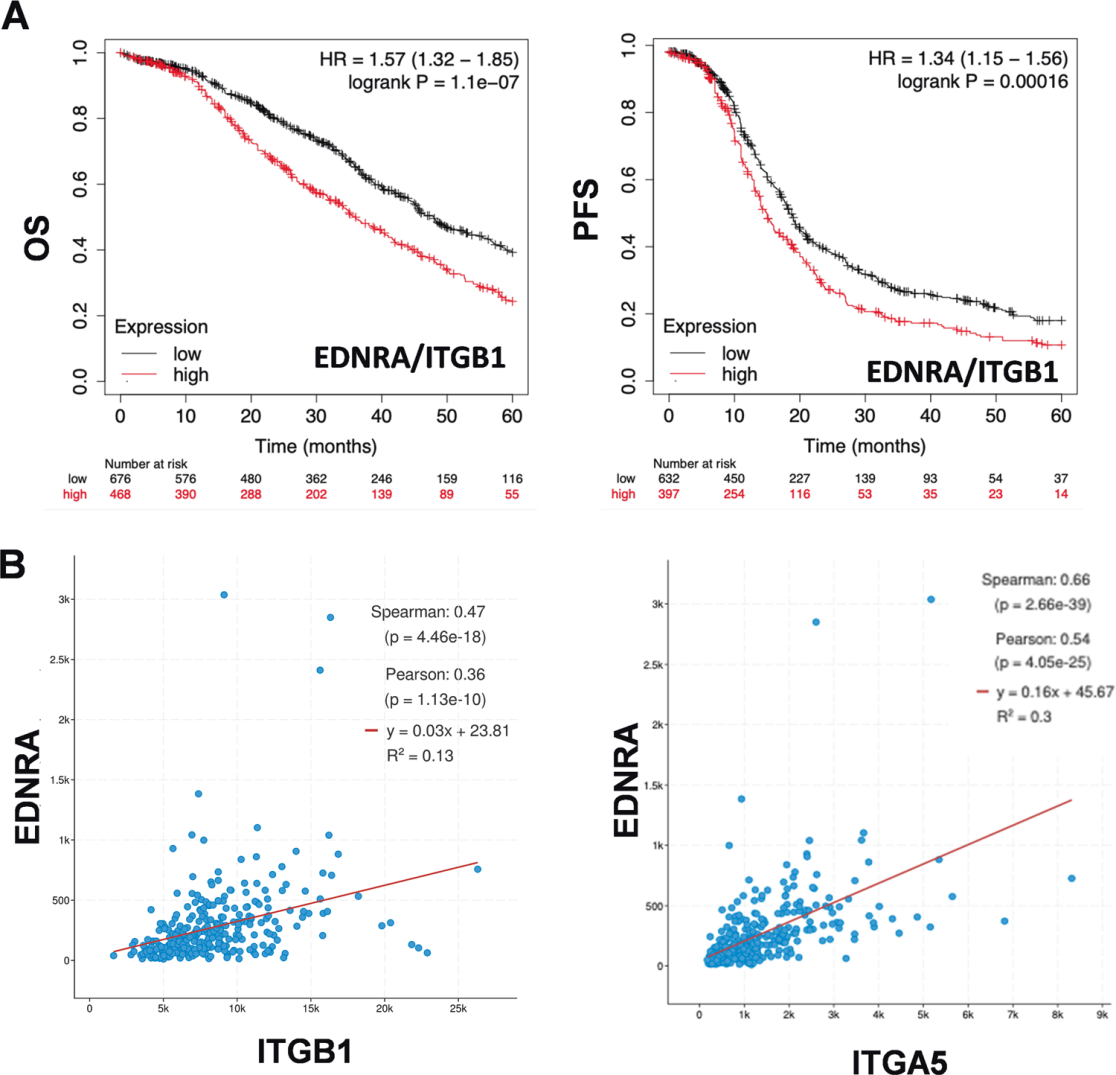
High ITGB1/EDNRA expression correlates with the HG-SOC poor prognosis. **A** Kaplan–Meier analysis of overall survival (OS) and progression-free survival (PFS) curves in HG-SOC patients with low or high ET_A_R (EDNRA)/Intβ1 (ITGB1) expression. **B** Dot plots from TCGA data illustrate the correlation between the endogenous EDNRA level with ITGB1 and ITGA5 mRNA levels.

To assess the biological significance and the functional effects of the interaction between Intβ1 and ET_A_R signaling, we first explored the existence of any correlation between the expression levels of Intβ1 (ITGB1) and ET_A_R (EDNRA) and their predictive value for HG-SOC prognosis. We used the online survival analysis software Kaplan–Meier plotter to generate survival curves and the log-rank test to evaluate the median expression values of combined high or low ITGB1 and EDNRA mRNA expression. As shown in Fig. 1A, the survival rate of patients with high ITGB1/EDNRA expression, in terms of overall survival (OS) and progression-free survival (PFS), was significantly worse than that of the low-expression group, supporting the idea that ITGB1/EDNRA expression could be used as a prognostic marker. We also evaluated ITGB1 and EDNRA co-expression using cBioPortal. Regression analysis revealed that their expression levels were positively correlated, indicating that ET_A_R may be related to the Intβ1 pathway in this tumor (Fig. 1B). In addition, a positive correlation between EDNRA and ITGA5 (Intα5) was evident in the same cohort (Fig. 1B).

### ET-1 regulates inside-out Intβ1 signaling

**Fig. 2:**
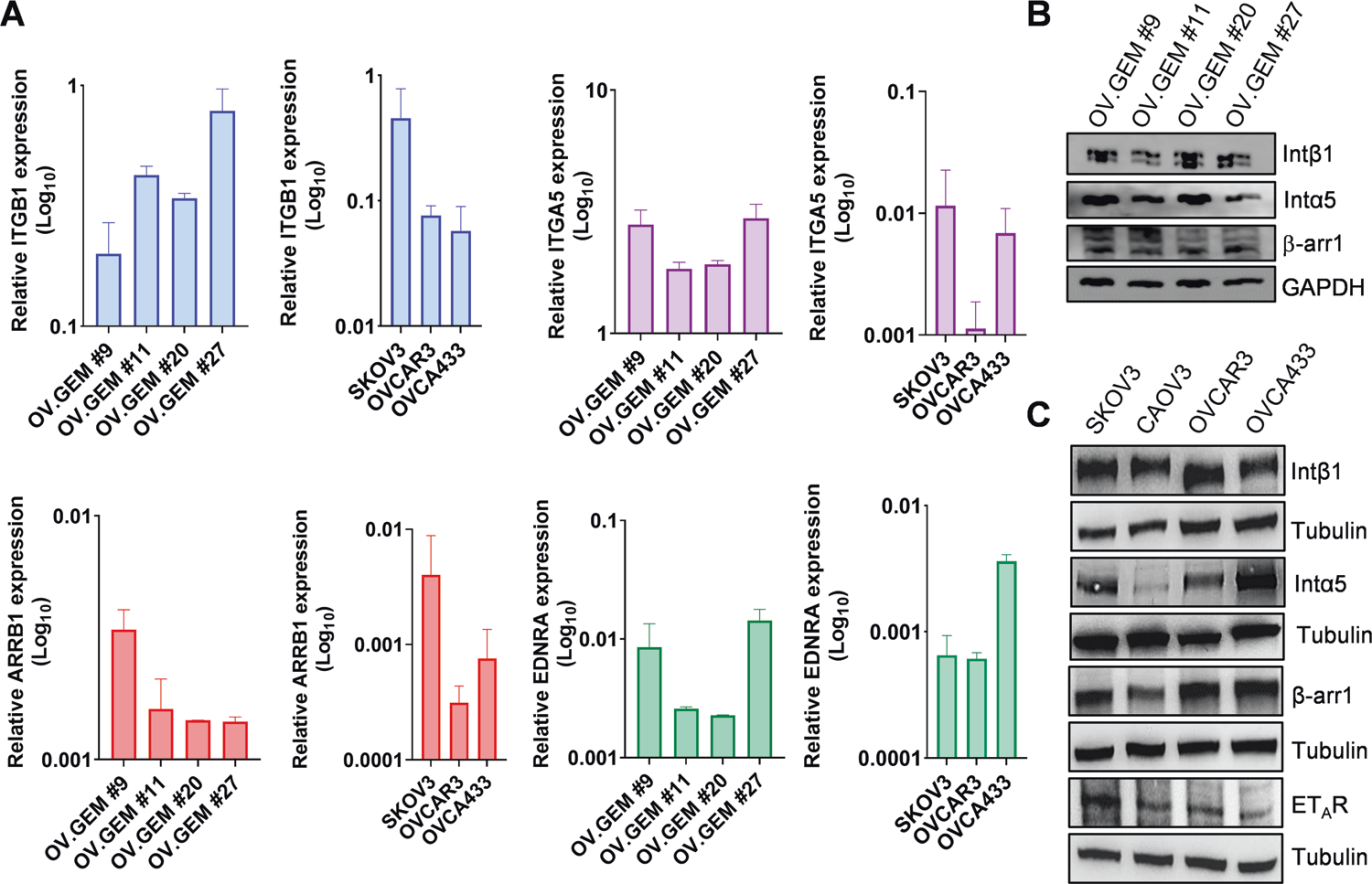
Expression of integrins, β-arr1 and ET_A_R. **A** qRT-PCR analysis for the expression of ITGB1 (Intβ1), ITGA5 (Intα5), ARRB1 (β-arr1), and EDNRA (ET_A_R) in a panel of primary HG-SOC cells (OV.GEM#9 and OV.GEM#11 from ovarian cancer tissues, OV.GEM#20 from peritoneal cancer tissue and OV.GEM#27 from omental cancer tissue) and cell lines. Representative WB analysis of indicated proteins in (**B**) primary HG-SOC cells and (**C**) cell lines.

**Fig. 3:**
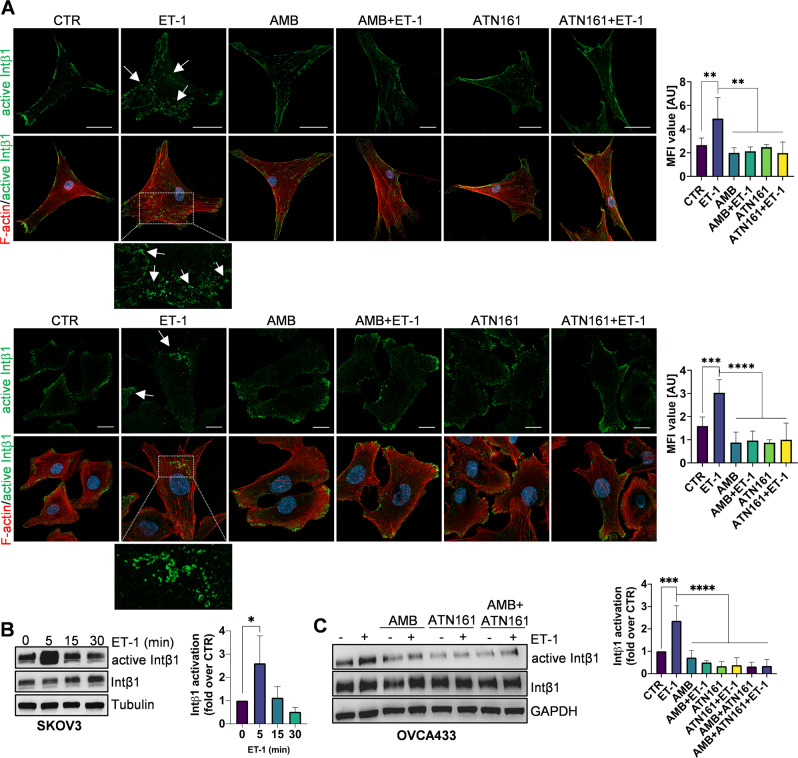
ET-1/ET_A_R/β-arr1 activates Intβ1 signaling. **A** CLSM analysis in OV.GEM#20 (upper) and SKOV3 (lower) cells, stimulated with ET-1 (100 nM) for 5 min and/or AMB (1 μm) and/or ATN161 (1 μm), stained for active Intβ1 (green) and F-actin (red). Nuclei are reported in blue (DAPI). For active Intβ1 a higher-power magnification image of a selected ROI in ET-1-stimulated cells is shown, indicating active Intβ1 intracellular accumulation. Scale bar, 50 μm. Histograms, mean fluorescence intensity (MFI) of active Intβ1/cytoplasmic area means ± SD. One-way ANOVA. **B** Lysates of cells stimulated with ET-1 for indicated times or (**C**) with ET-1 for 5 min and/or AMB and/or ATN161 were subjected to WB for indicated proteins. Histograms, means ± SD of the average band intensity normalized to Tubulin or GAPDH (fold changes versus CTR) used as loading control; *n* = 3, one-way ANOVA.

**Fig. 4:**
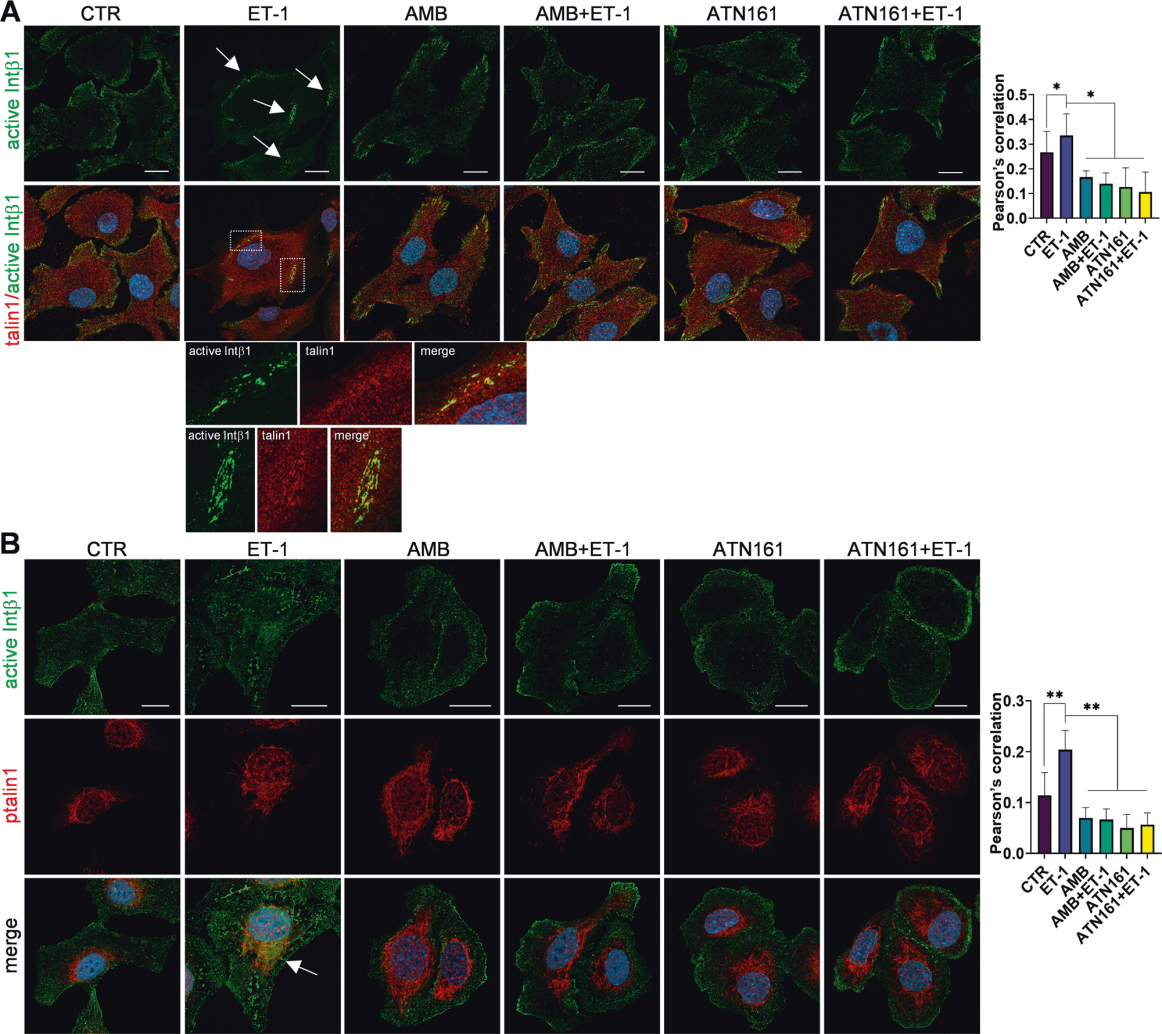
ET-1 promotes the phosphorylation of talin1 and its association with active Intβ1. **A** CLSM analysis of SKOV3 cells stimulated with ET-1 for 5 min and/or AMB and/or ATN161, then stained for active Intβ1 (green) and talin1 (red) detection. Colocalization is shown in merged images, detected in yellow. For ET-1 stimulation, intracellular accumulation of active Intβ1 is depicted by arrows and higher-power magnification images of two selected ROI are shown, bringing out active Intβ1/talin1 colocalization. Nuclei are reported in blue (DAPI). Scale bar, 20 μm. Columns show the mean ± SD of quantification of Pearson’s correlation between active Intβ1 and talin1. **B** CLSM analysis of SKOV3 cells stimulated with ET-1 and/or AMB and/or ATN161 for 5 min and stained for active Intβ1 (green) and ptalin1 (red). Colocalization is shown in merged images, detected in yellow and depicted by an arrow. Nuclei are reported in blue (DAPI). Scale bar, 20 μm. Columns, mean ± SD of quantification of Pearson’s correlation between active Intβ1 and ptalin1. *n* = 3, one-way ANOVA.

To dissect the molecular mechanisms linking ET_A_R and Intβ1 signaling, we used a panel of cells, including primary HG-SOC cells (derived from HG-SOC ovarian, omental, and peritoneal tissues) [21] and commercial cell lines, representing SOC (SKOV3, OVCA433) or HG-SOC cells (OVCAR3, CAOV3). Since the binding of Intα5β1 to the peritoneum/omentum contributes to the initial adhesion/invasion and later helps cancer cells to metastasize [22], we measured Intβ1, Intα5, β-arr1, and ET_A_R expression using qPCR and western blotting (WB). All HG-SOC primary cells expressed Intβ1, Intα5, β-arr1, and ET_A_R at different levels (Fig. 2A, B). All cell lines expressed high levels of Intβ1, while SKOV3, OVCA433, and OVCAR3 cells expressed high levels of Intα5 (Fig. 2A, C). Considering that GPCRs might modulate integrin affinity and activity by inside-out signaling [7], we investigated whether and how ET_A_R activation by ET-1 regulates Intβ1 signaling by employing a conformation-specific antibody that recognizes its active form (9EG7). Confocal laser scanner microscopy (CLSM) analysis showed significant Intβ1 activation and intracellular accumulation upon ET-1 addition (Fig. 3A and Supplementary Fig. 1A). WB analysis confirmed Intβ1 activation and downstream FAK and paxillin signaling driven by ET-1 (Fig. 3B, C and Supplementary Fig. 2A–E). This effect is linked to ET_A_R activation, as demonstrated by the inhibitory effect of AMB, a specific ET_A_R antagonist, to the same extent as ATN161, a small peptide antagonist of Intα5β1, or Intβ1 silencing (Fig. 3A, C and Supplementary Fig. 1A), demonstrating that ET-1 and Intβ1 are acting to affect the same functional pathway. The combined treatment of AMB + ATN161 showed a slight but not significant increase compared to single drugs (Fig. 3C). Co-staining of paxillin-GFP with phalloidin demonstrated that ET-1 treatment was consistent with a marked increase in focal adhesion (FA) formation (Supplementary Fig. 2F). All these findings, together with enhanced cell adhesion (Supplementary Fig. 1B), indicate the presence of an inside-out intβ1 signaling driven by ET-1. As talin1 is required for inside-out integrin activation [23–25] and is expressed in SOC cells (Supplementary Fig. 3A), we assessed whether talin1 contributes to Intβ1 activation. CLSM analysis showed the recruitment of talin1 to active Intβ1 in ET-1-stimulated cells, but not in cells treated with AMB or ATN161 (Fig. 4A). The silencing of talin1 reduces paxillin activation (Supplementary Fig. 2E), demonstrating that talin1 is responsible for ET-1-driven Intβ1 signaling. Since talin1 phosphorylation at S425 is associated with some integrin functions [25], we examined talin1 phosphorylation and found enhanced talin1 phosphorylation in ET-1-treated cells compared to control cells, which was inhibited by treatment with AMB or ATN161 (Fig. 4B and Supplementary Fig. 3B, C). Moreover, CLSM analyses showed enhanced localization of active Intβ1 with phosphorylated talin1 in the presence of ET-1, but not AMB or ATN161 (Fig. 4B and Supplementary Fig. 3C), indicating that ET-1 might induce Intβ1 activation via talin1 phosphorylation.

### ET-1-dependent β-arr1 interaction with Intβ1 and talin1 regulates Intβ1 signaling

**Fig. 5:**
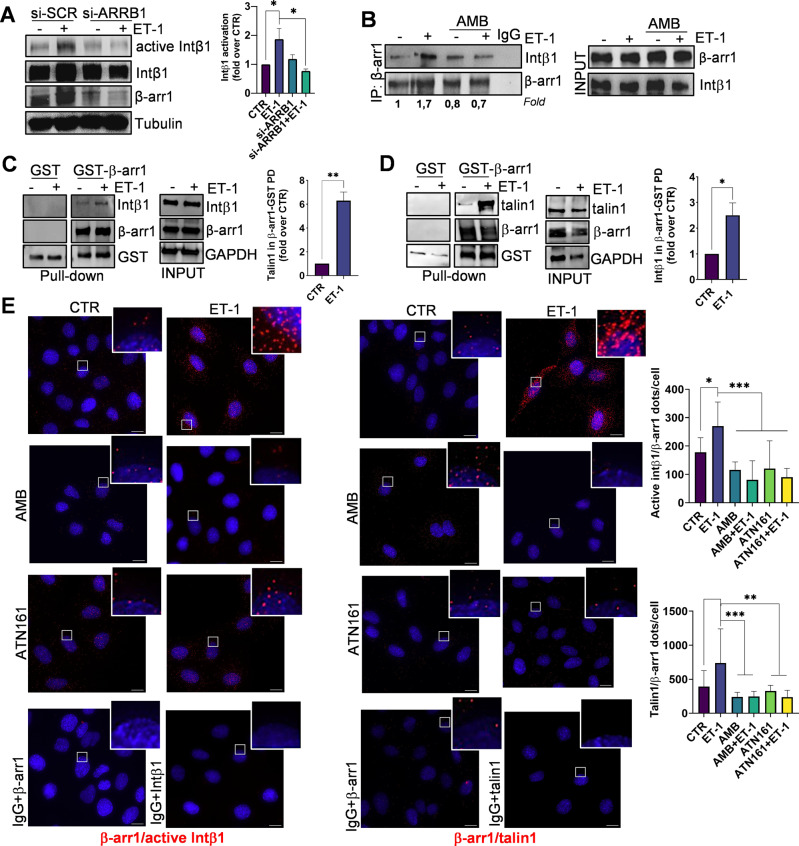
β-arr1 links Intβ1 and talin1. **A** si-SCR and si-ARRB1 transfected SKOV3 cells, stimulated with ET-1 for 5 min, were subjected to WB for indicated proteins. Histograms mean ± SD of the average band intensity normalized to Tubulin used as a loading control (fold changes versus CTR); *n* = 2, one-way ANOVA. **B** Lysates of SKOV3 cells stimulated with ET-1 and/or AMB for 5 min were immunoprecipitated (IP) with anti-β-arr1 or irrelevant IgG. **C**, **D** Lysates of OVCAR3 cells stimulated or not with ET-1 were incubated with GST or GST-β-arr1 fusion protein. Representative images of inputs and pulldown analyzed by WB for indicated proteins. Histograms, means ± SD of the average band intensity normalized to GAPDH used as a loading control (fold changes versus CTR); *n* = 2, *t* test. **E** Representative PLA images of protein complexes containing Intβ1 and β-arr1 or talin1 and β-arr1 in OVCAR3 cells stimulated with ET-1 and/or AMB and/or ATN161 for 60 min. The red signal represents a positive PLA reaction and DAPI staining (blue) highlights the nucleus. No positive PLA reaction was observed in negative controls (with primary antibodies and irrelevant IgG). Scale bar, 10 μm. Inset, show higher magnifications of the square. Histograms mean ± SD of PLA dots per nucleus; *n* = 3. One-way ANOVA, Tukey post hoc analysis.

We recently demonstrated a link between ET_A_R and integrin-related signaling dependents on β-arr1, which is involved in cytoskeletal remodeling and cell motility [17]. As β-arr acts as a modulator of GPCR signaling to integrins [26, 27], we tested the involvement of β-arr1 in ET_A_R-dependent Intβ1 signaling, demonstrating that silencing of β-arr1 significantly inhibited Intβ1 activation (Fig. 5A). Different approaches have been used to evaluate whether β-arr1 interacts with Intβ1 or talin1. As shown by co-IP assays and GST pull-down assays, ET-1 promotes the association of β-arr1 with Intβ1 or talin1, but not in the presence of AMB (Fig. 5B–D and Supplementary Fig. 4B). These interactions were also confirmed by PLAs between endogenous Intβ1 with talin1 or β-arr1 (Fig. 5E, and Supplementary Fig. 4A), establishing Intβ1/talin1 as novel interactors of β-arr1 driven by ET-1/ET_A_R. Silencing of talin1 impaired the interaction between β-arr1 and Intβ1 induced by ET-1 (Supplementary Fig. 4B), whereas silencing of β-arr1 inhibited the interaction of talin1 with Intβ1 (Supplementary Fig. 4C). Moreover, CLSM analyses showed that ET-1-dependent localization of active Intβ1 with phosphorylated talin1 was inhibited in cells silenced for β-arr1 (Supplementary Fig. 3C). Altogether, these findings demonstrate a key role for β-arr1 in favoring the interaction of talin1/Intβ1 and in sustaining ET-1/ET_A_R-dependent Intβ1 signaling.

### ET_A_R/β-arr1/Intβ1 signaling supports spheroid survival and their mesothelium-intercalation capacity

**Fig. 6:**
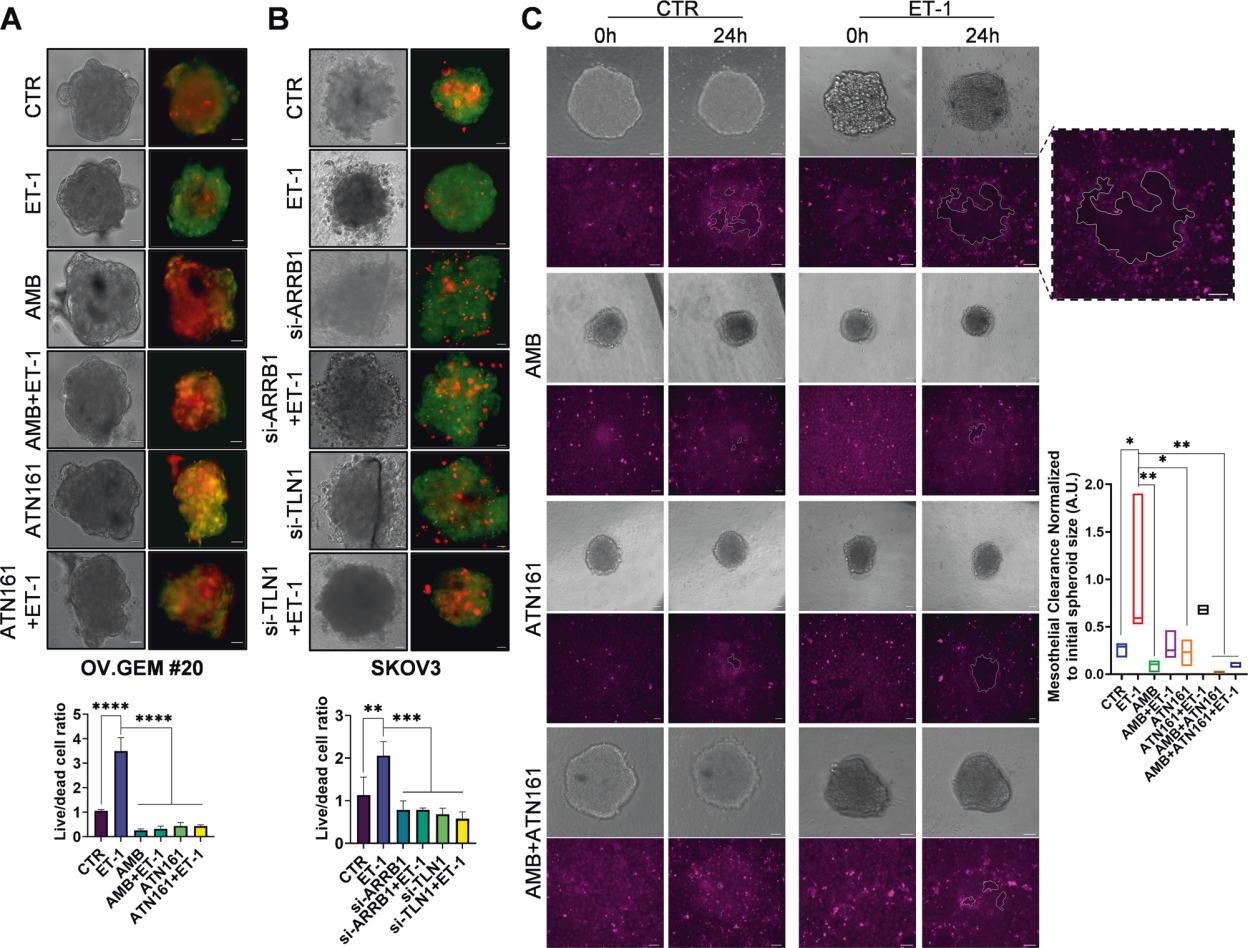
HG-SOC cell spheroid survival and mesothelial clearance are regulated by ET-1/ β-arr1/Intβ1 signaling. **A** 3D spheroids were treated with ET-1 and/or AMB and/or ATN161 for 72 h or (**B**) si-SCR, or si-ARRB1 or si-TLN1 transfected 3D spheroids were treated with ET-1 and live (green) or dead (red) cells were determined using a dual-fluorescence system. Histograms mean ± SD of the live/dead cell ratio (fold changes versus CTR); *n* = 2, one-way ANOVA. **C** Images depict mesothelial clearance induced by SKOV3 spheroids treated with ET-1 and/or AMB + ATN161 at 0- and 24-h time points. Scale bar, 50 μm. The graph represents the ratio between the area of the “hole”/aperture in the mesothelial monolayer after 24 h (highlighted with the white line) and the initial spheroid area (0 h). *n* = 2, one-way ANOVA.

Having demonstrated that the ET-1-dependent signal might accomplish the interaction between SOC cells and MCs in promoting stromal invasion [17] and considering the role of integrin/talin1 in mesothelial clearance [22], we tested whether ET_A_R/β-arr1-dependent Intβ1 signaling might support MC displacement. We first tested the adhesion of cancer cells to MCs grown on fibronectin and found that ET-1 promoted cell adhesion, which was significantly inhibited by treatment with AMB or ATN161 or in combination (Supplementary Fig. 5A). Moreover, silencing of talin1 inhibits ET-1-dependent trans-mesothelial migration of cancer cells (Supplementary Fig. 5B). Since SOC spheroids represent metastatic units that invade the basement membrane and Intβ1 controls their invasive capacity [28–32], we evaluated the role of ET_A_R/Intβ1 in promoting the spheroid formation and mesothelium-intercalation capacity. Primary HG-SOC cells, OVCA443 and SKOV3 cells plated on low-adherence plates aggregated and formed spheroids. Live/dead assays demonstrated that ET-1 enhanced the number of live cells in spheroids compared to that induced in the presence of AMB or ATN161 or combination (Fig. 6A and Supplementary Fig. 6). Furthermore, the inhibitory effects of silencing of β-arr1 or talin1 (Fig. 6B) demonstrated that ET-1-dependent talin1/Intβ1 signaling induces cell survival in multicellular clusters. To investigate the interaction between SOC spheroids and MCs, we used OVCA433 and OV.GEM#20 cell spheroids and fluorescently labeled MCs (red or Cy5) and followed the dynamics of a mesothelial monolayer after cancer spheroid attachment by live confocal analysis. The clearance area increased when ET-1 was added to the spheroids (Fig. 6C, Supplementary Fig. 7A, C, and Supplementary Videos S1 and S2), but this effect was inhibited in the presence of AMB or ATN161, or in combination (Fig. 6C and Supplementary Videos S3–S8). Cancer cell adhesion to an MC monolayer involves integrins and the linkage to the actin and myosin network through the recruitment of talin1 [22]. Since Intα5β1 facilitates the generation of contractile forces that enhance the invasiveness of spheroids [30, 31] and contractile morphology is associated with Rho activity, we examined whether ET-1-dependent Intα5β1 might regulate contractile machinery to promote mesothelial clearance. According to previous data [16, 33], ET-1 activates RhoA GTPase, and this effect is lost upon silencing of Intβ1 (Supplementary Fig. 7C), suggesting an indirect effect although we cannot exclude also a possible direct involvement of Intβ1 in ET-1-dependent RhoA activation. We also performed mesothelial clearance in the presence or absence of the Rho kinase (ROCK) inhibitor Y27632 (10 μM) and found that Y27632 significantly reduced the ET-1-induced effect (Supplementary Fig. 7A and Supplementary Videos S9 and S10). Our data suggest that the engagement of Intα5β1 is an important step in spheroid-induced mesothelial cells and requires the linkage of integrins to the actomyosin network for ET-1-dependent mesothelial clearance.

### ET-1/β-arr1/Intβ1 signaling regulates cell invasion in the organotypic model

**Fig. 7:**
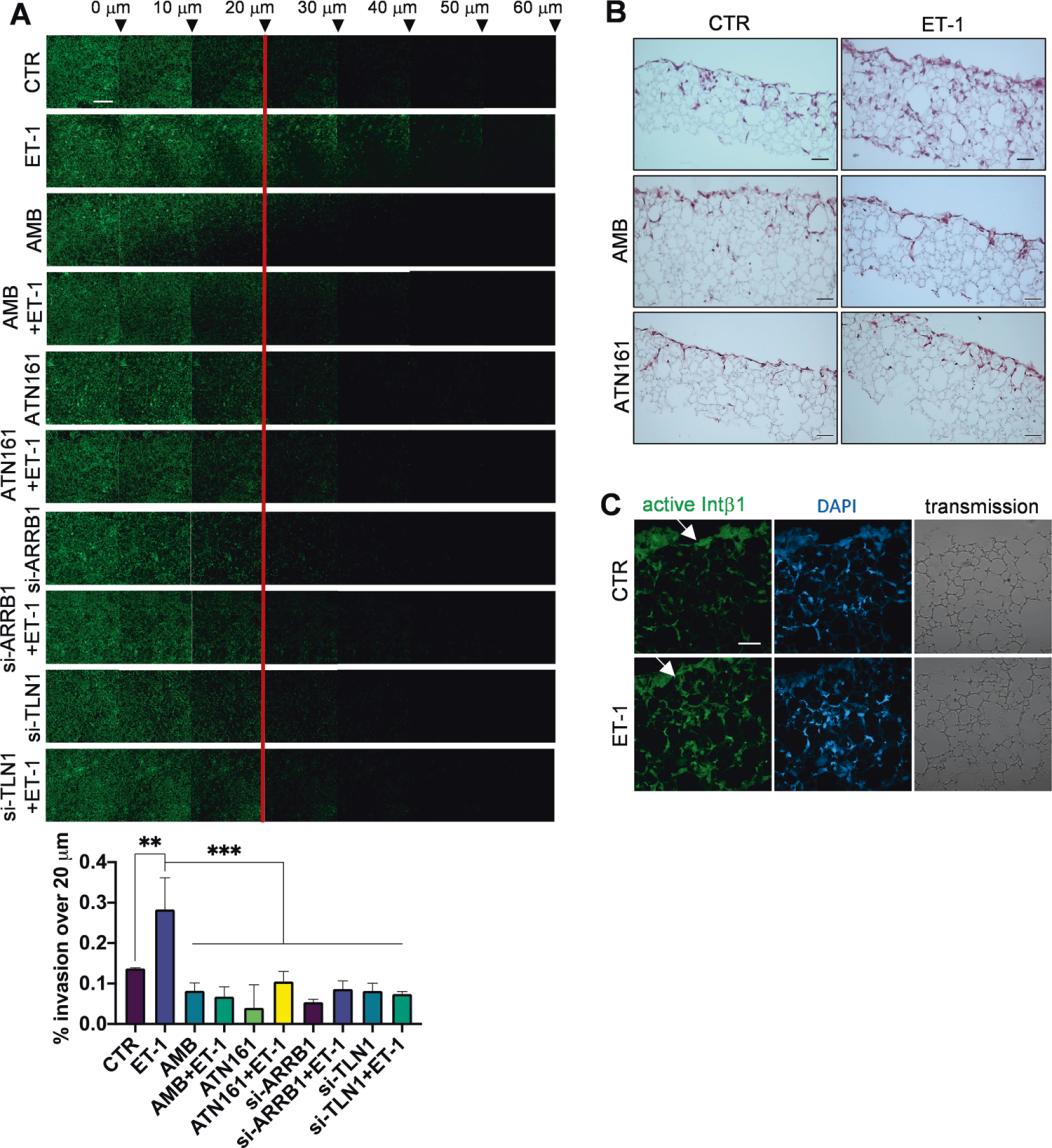
HG-SOC invasion is regulated by ET-1/Intβ1 signaling. **A** si-SCR, or si-TLN1 or si-ARRB1 OVCAR3 cells treated with ET-1 and/or AMB and/or ATN161 were allowed to invade fibronectin/type I collagen plugs in an inverted invasion assay (48 h). Cells were stained with PKH67, and serial optical sections (10 μm intervals) were acquired. The invasion was measured by dividing the sum of signal intensity of all slides beyond 20 μm (invading cells) by the sum of the intensity of all slides (total cells). *n* = 2, one-way ANOVA, Tukey post hoc analysis. Scale bar, 200 µm. **B** SKOV3 cells plated on a monolayer of MCs grown on fibronectin/type I collagen in a polystyrene scaffold were allowed to invade for 7 days in the absence and presence of ET-1 and/or AMB and/or ATN161, then fixed with Bouin’s solution and paraffin-embedded scaffolds were then cut into thin slices (10 µm). The images show cell invasion in a 3D organotypic model. Hematoxylin and eosin staining are shown. **C** Sections as in (**B**) were stained for active Intβ1 (green) and DAPI (blue) detection. The corresponding transmitted light images are also shown. Arrows depict the top side of the scaffold where cells were plated. Scale bar, 50 µm.

To assess the requirement of ET-1-dependent Intα5β1 signaling for the ability of cancer cells to invade, we performed a test using a 3D matrix invasion assay in which cancer cells were seeded on top of a 500-μm thick containing fibronectin/type I collagen gels. Cells adhered to and invaded these gels, and the addition of ET-1 significantly enhanced the average invasion depth into the ECM, whereas this effect was almost lost when cells were treated with AMB, ATN161 and upon silencing of talin1 or β-arr1 (Fig. 7A). To study these effects in a model that replicates the omental premetastatic niche, we developed an organotypic model by using a polystyrene scaffold engineered into a 200-µm thick membrane which provides a 3D space into which cells can invade, proliferate, and grow. After coating the scaffold with fibronectin and MCs, cells were able to attach, grow, and colonize the full thickness of the scaffold within 7 days of the addition of ET-1, but not in the presence of AMB or ATN161 (Fig. 7B). Immunocytochemical analysis confirmed the expression of active Intβ1 in ET-1-treated SOC cells invading the matrix (Fig. 7C). Taken together, these findings support the idea that the ET_A_R/Intα5β1 axis enhances the invasive potential of SOC cells.

### Ambrisentan as well as ATN161 controls SOC cell metastatic colonization

**Fig. 8:**
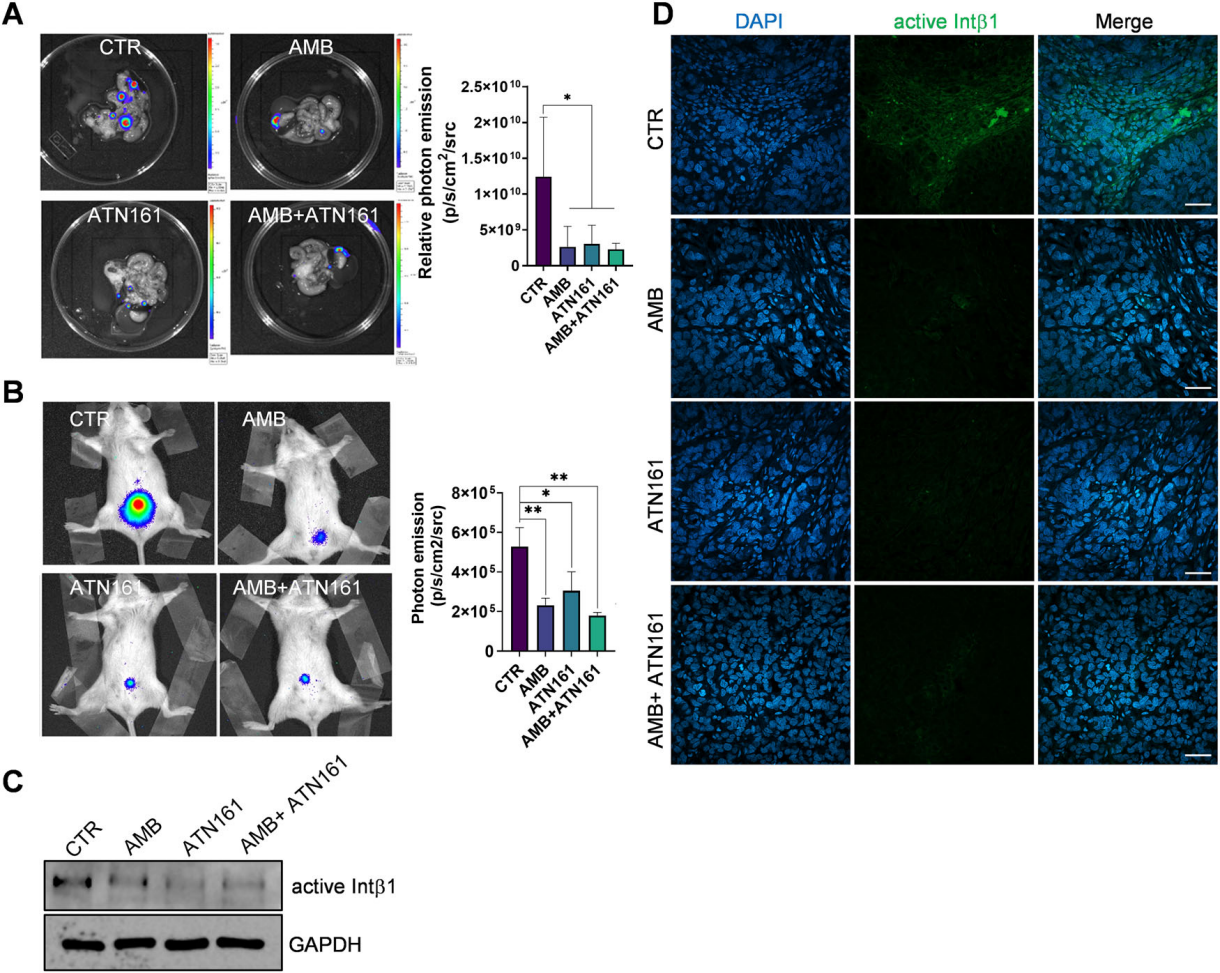
Ambrisentan as well as ATN161 controls SOC cell metastatic colonization . **A** In vivo adhesion assays as showed by bioluminescent images of SKOV3-Luc cells, untreated (CTR) or pretreated with AMB or ATN161 or with a combination, on abdominal organs (*n* = 5 mice/group). The organs arranged are the intestine and mesentery, the spleen with the pancreas, and omentum. The adherent cell aggregates were observed, and the luminescence was read and recorded. *n* = 2 one-way ANOVA. **B** Bioluminescent images of intraperitoneally (i.p.) injected SKOV3-Luc cells (2 × 10^6^ cells) in NOD/SCID mice, undergoing treatments for 5 weeks with 200 L Metocell (vehicle, CTR) or 200 μL AMB (10 mg/kg, oral daily), both by oral gavage, or ATN161 (100 μg/kg, i.p. twice a week). Tumor burden was assessed on days 17, 25, 32, and 39 after tumor cell injection. Data are presented as mean ± SD, *n* = 2, one-way ANOVA. **C** Representative WB for Intβ1 expression in metastatic nodules. GAPDH was used for loading control. **D** Representative CLSM images of paraffin-embedded formalin-fixed tumor tissue sections stained for active Intβ1 from mice as in (**B**), stained for active Intβ1 (green). Nuclei in the tissue sections were counterstained with DAPI. Scale bar = 50 µm.

The results of our in vitro studies suggest that ET_A_R/Intα5β1 contributes to the metastatic potential of SOC cells by regulating anchorage-independent cell survival, invasion, and mesothelial clearance. To translate our in vitro findings into an in vivo model, we assessed the extent to which the blockade of ET_A_R or Intα5β1 might inhibit tumor adhesion and metastatic dissemination. We used intraperitoneally injected SKOV3-Luc cells in mice to mimic SOC cell seeding on peritoneal surfaces, as observed in patients with advanced stages of the disease. For in vivo adhesion assays, untreated or pretreated tumor cells with AMB or ATN161, or in combination, were i.p. injected, and abdominal organs were excised. Based on the ex vivo bioluminescence (BLI) value from luciferase-expressing cells, significantly more tumor cells were attached to the intraperitoneal organs in the control group than in the single drug-treated group or when drugs were combined (Fig. 8A). To test the effect of drugs on intraperitoneal dissemination, mice were treated with vehicle, AMB, ATN161, or both, for 5 weeks, and tumor cell propagation in the peritoneal cavity of mice was evaluated by BLI images, demonstrating that either AMB or ATN161 or their combination significantly inhibited intraperitoneal spreading (Fig. 8B). The peritoneal metastatic organs were examined, and several nodules were recorded and used for WB and CLSM analyses. Reduced expression of active Intα5β1 was evident in tissues from AMB or ATN161 or ATN161 + AMB-treated mice (Fig. 8C, D). Collectively, these results indicate that blocking ET_A_R and Intα5β1 is effective to control Intβ1-dependent signaling during metastatic colonization.

## Discussion

With the growing success in treating HG-SOC in the clinical setting parallels to the development of new target therapies based on molecular characterization of these tumors, there is a constant push to expand our knowledge on how this tumor invades and metastasizes [34]. The intraperitoneal route of ovarian cancer metastasis places unique demands on tumor cells, such as single or spheroids, requiring specific molecular mechanisms [35, 36]. Cues from the ECM, heterotypic interactions between tumor and stromal cells, and growth factor signaling are drivers of these processes [37]. Among these, the quality and strength of integrin-mediated signaling depend not only on the local ECM density/stiffness and cell contractility but also on the cell-intrinsic signaling network working in concert to steer the invasive machinery [2, 3]. The data in this study provide evidence of a novel mechanism of integrin activation driven by ET_A_R/β-arr1 supporting the metastatic process. Using primary HG-SOC cells and cell lines, we found that (1) ET-1/ET_A_R promotes Intα5β1 inside-out signaling via a functional interaction between β-arr1/Intβ1 and talin1; (2) this signaling enhances spheroid survival and mesothelial clearance competence with increased cellular contractility and supports cell invasion; (3) targeting of ET_A_R as well as Intα5β1 significantly inhibited adhesion and spreading to intraperitoneal organs, and activation of Intβ1; (4) the co-expression of ET_A_R and Intβ1 in patients with HG-SOC correlated with poor prognosis, thus representing potential biomarkers of metastatic progression.

Integrins are critical mechano-signal transducers mediating the effects of ECM and cellular receptor signaling during tumor progression [7, 38]. One aspect of their function is linked to the “inside-out” activation, which places tumor cells at a relative advantage in the metastatic process [2, 3]. Previous studies established the primacy of Intβ1 function in determining the success of ovarian cancer cell metastatic colonization, with a high tropism for intraperitoneal organs with a monolayer of MCs covering the underlying stroma [8]. Although the role of Intα5β1-fibronectin interaction has been described [8], the contribution of inside-out signaling has not been fully elucidated. Signals from GPCRs may lead to integrin subunit phosphorylation and inside-out integrin activation [4, 6]. Indeed, the interaction of GPCR-dependent Gα_13_ or Gβγ with Intβ1 or Intβ3 regulates integrin-mediated cell spreading and migration. In this context, chemokine receptors, protease-activated receptors, and T-cell receptor engagement are well-recognized triggers for integrin activation [7, 39–41]. Previous studies have demonstrated that β-arr might modulate GPCR signaling to integrins, acting downstream of chemokine-triggered Gα_i_ activation [26]; however, the interaction with specific GPCRs and related proteins remains to be defined.

Our study demonstrates that activated ET_A_R shifts Intβ1 into a high-affinity conformation via inside-out activation, accelerating cell spreading and migration, and further supports the role of GPCRs in the regulation of Intα5β1-linked cancer invasion and metastasis. Moreover, we provide evidence that this process is linked to the β-arr1 function, a recognized interactor of cytoskeletal remodeling proteins [42]. Previous data have demonstrated that β-arr2 is required for integrin-mediated leukocyte adhesion during CXCR2-driven extravasation [26] and acts as a specific regulator of epithelial cell adhesion, FA formation, and Intα5β1 trafficking downstream of type III TGF-β receptor [43]. In addition, β-arrs, which link microtubules and clathrin, are essential for endocytic machinery to properly target FAs and internalize integrins [26]. Although our previous findings demonstrated that β-arr1 is associated with ILK within the integrin signaling [17], these data highlight a new function of β-arr1 involving talin1, which is recognized to play a key role in the first step of integrin activation. However, its recruitment to integrins seems to be cell type-and/or tissue-specific and remains to be fully clarified [41, 44]. As in our cellular model β-arr1 can directly bind both Intβ1 and talin1, we speculate that these interactions accelerate the activation process. Of note, ET-1 enhances the phosphorylation of talin1, which is predicted to promote an “open” conformation and facilitate Intβ1 activation.

We propose a new modality of integrin-specific signaling activation independent of ECM ligands. This allows cancer cells to adapt to the ECM properties and to invade and metastasize, where the control of talin1 recruitment and integrin activation adds to the diversity of cellular responses that influence tumor progression.

These findings integrate and expand our previous work demonstrating that ET-1/β-arr1 favors the interaction between SOC cells and MCs [17, 45]. The upstream activation of Intα5β1 within multicellular aggregates may contribute to pro-survival signaling and is linked to adhesive and clearance-competent cancer spheroids in the mesothelial monolayer. According to previous data demonstrating the role of Intα5β1 in spheroid formation/survival and mesothelial clearance [22, 28, 29], our findings further support the idea that the abundance of active Intα5β1 in ovarian cancer cells contributes to perturbing the mesothelial barrier and enhances the invasive behavior of cancer cells, allowing them to penetrate the basement membrane and gain access to the stroma for the formation of secondary tumor growth.

The complex biochemical and mechanical regulation of integrin function in influencing cancer progression has led to the design in the last years of potential integrin-based therapies, including drugs targeting Intα5β1 [2, 46]. Recent evidence demonstrated a role for Intβ1 cytoplasmic tail in facilitating cancer lung cell malignant phenotype through ECM-binding-independent signaling [47]. These findings also highlight that anti-integrin cancer therapies targeting the cytoplasmic tail and its interactions might be successful, also in cooperation with drug-disrupting integrin-ECM interactions. Therefore, to improve the efficacy of integrin-targeted therapy, new strategies targeting integrins and effectors, or combined with other targeted therapies may be tested.

To test the therapeutic relevance of our data, we took advantage of a 3D organotypic model, which is considered an excellent platform to reconstruct the organ-specific TME, to understand the influence on metastatic features, and to investigate new therapeutics and their responses [48]. Using this model, we tested the therapeutic anti-metastatic potential of AMB, a selective ET_A_R antagonist approved by the FDA and EMA for the treatment of pulmonary hypertension, or ATN161, highlighting the advantage of these approaches. AMB is effective in interfering with ET_A_R/β-arr1 and inhibiting both adhesions to intraperitoneal organs and the metastatic potential of SOC cells, in a way comparable to ATN161, indicating that ET-1 signaling acts on the same function pathway of Intβ1. The combination therapy, although a difference is observed, is not significantly effective compared to single drugs. However, we speculate that different therapeutic strategy treatments (with different integrin-targeting agents or early treatment) must be tested to finally verify the effects of combinatorial treatments.

Interestingly, bioinformatic analyses showed that high expression levels of ET_A_R/Intβ1 were positively correlated with poor prognosis, representing potential predictive prognostic markers in patients with HG-SOC. Collectively, these data establish a novel interaction between ET_A_R/β-arr1 and Intβ1 signaling to direct cell invasive behavior in the ovarian premetastatic niche and suggest that in the new integrin-targeting programs the interaction of Intα5β1 with ET_A_R/β-arr1 is an important part of this process to be explored.

## Materials and methods

### Generation of primary cancer cells from patient tissues, culture, and characterization

This study included four patients with newly diagnosed, histologically confirmed HG-SOCs admitted to the Gynecologic Oncology Unit, Fondazione Policlinico Universitario A. Gemelli, IRCCS, Roma, between July 2020 and September 2021. The study was approved by the local ethics committee and institutional review board (Protocol 19402/18 ID:2045) and all patients provided written informed consent. All the data were managed using anonymous numerical codes (OV.GEM). Patient characteristics are reported in Supplementary Table S1.

The experimental procedures related to primary cancer cell culture and characterization have been described previously [49]. Briefly, samples were collected at surgery from “leftover tissues” by using sterile scalpels. Tumor specimens were finely minced into small fragments using surgical blades and then enzymatically digested using the Tumor Dissociation Kit (Miltenyi Biotec Bologna, Italy), according to the manufacturer’s protocol. The cell suspension was applied to a MACS SmartStrainer (70 μm), placed in a tube, washed, centrifuged, and resuspended in a complete DMEM/F12 (1:1) medium, supplemented with heat-inactivated fetal bovine serum (10%), glutamine (2 mM), and kanamycin (2 mM) (Life Technologies, CA, USA). Cells were maintained at 37 °C in the presence of 5% CO_2_ and 95% humidified air. The medium was changed 48/72 h after the initial plating and every 3 days thereafter. Stromal cells, primarily fibroblasts, progressively disappeared during subcultivation. Each patient-derived cell line was characterized by morphology and immunocytochemistry. To determine the purity of the cells following isolation and culturing, cytospin preparations were stained with anti-cytokeratin 7 (Clone OV-TL 12/30, Agilent Dako, Santa Clara, CA USA, ready-to-use) and anti-human fibroblasts (clone TE-7, Sigma-Aldrich, Merck KGaA, Darmstadt, Germany dilution 1:100). The selected cell lines had typical primary epithelial culture morphology; their epithelial nature was confirmed by cytokeratin 7 staining, and the absence of stromal fibroblast overgrowth was verified by TE-7 staining.

### Cell line cultures

The human ovarian serous adenocarcinoma cell lines SKOV3 (ATCC® HTB-77™), OVCAR3 (ATCC® HTB-161™), and CAOV3 (ATCC® HTB-75™) were obtained from the American Type Culture Collection (LGC Standards, Teddington, UK). OVCA433 (RRID: CVCL_0475) was provided by Prof. G. Scambia (Catholic University School of Medicine). SKOV3 cells were maintained in McCoy’s 5A medium (Cat# 26600-023; GIBCO Thermo Fisher), OVCAR3 in RPMI-1640 medium (Cat# 618700-010; GIBCO Thermo Fisher), OVCA433, and CAOV3 in Dulbecco’s modified Eagle medium (Cat# 21885-025; GIBCO Thermo Fisher).

Human primary mesothelial cells (Cat# DMES-F) were obtained from Zen-Bio Inc. (USA) and maintained in a mesothelial cell growth medium (Cat# MSO-1; Zen-Bio). All media were supplemented with 10% fetal calf serum, 50 U/mL penicillin, and 50 mg/mL streptomycin. Cells were incubated at 37 °C in a humidified atmosphere containing 5% CO_2_.

When appropriate, the cells were incubated in serum-free media with ET-1 (Cat# E7764-1MG; Sigma-Aldrich) at 100 nmol/L for the indicated times. Ambrisentan (AMB, also called (+)-(2S)-2-[(4,6dimethylpyrimidin-2-yl)oxy]-3-methoxy-3,3-diphenylpropanoic acid, Cat# SML2104; Sigma-Aldrich), ATN161 (Cat# 6058; Tocris), or Y27632 (10 μM), from Alexis Corporation (Enzo Life Sciences, Inc., Farmingdale, NY, USA), was used at a concentration of 1 μmol/L for 30 min before the addition of ET-1. All the cells were routinely tested for mycoplasma contamination.

### Antibodies and chemical reagents

Antibodies (Abs) used for Western blotting (WB) were as follows: anti-CD29 (9EG7) (Cat# 550531; RRID:AB_393729; BD Biosciences), anti-CD29 (Cat# 610468; BD Biosciences), anti-β-arr1 (Cat# ab32099; Abcam), anti-Tubulin (Cat# sc-32,293; RRID:AB_628412; Santa Cruz), anti-endothelin A receptor (Cat# PA3-065; Thermo Fisher), anti-phospho-talin1 (Ser425) (Cat# TP171; RRID:AB_2840569; ECM Biosciences), anti-talin1 (Cat# MA5-28133; RRID:AB_2204003; Invitrogen), anti-GAPDH (Cat# G945; RRID:AB_10597731; Sigma-Aldrich), anti-phospho-paxillin (Cat# ABP-0156; Immunological Sciences), anti-paxillin (Cat# MAB-80128; RRID:AB_11187804; Immunological Sciences), anti-phospho-FAK (Cat# ABP-0290; RRID:AB_2173671; Immunological Sciences), anti-FAK (Cat# MAB-10157, RRID:AB_10905163; Immunological Sciences), anti-GST (Cat# sc-138, RRID:AB_627677; Santa Cruz), anti-Integrin α5 [EPR7854] (Cat# ab150361; Abcam), anti-β-actin (Cat# A2228, Sigma-Aldrich), horseradish peroxidase-conjugated goat anti-rabbit (Cat# 32460, Life Technologies) or anti-mouse (Cat# PA128568, Life Technologies). The antibodies used were as follows: anti-β-arr1 (Cat# LSC156512; LSBIO), anti-CD29 (9EG7), anti-phospho-talin1 (Ser425), and anti-talin1. F-actin was visualized using Alexa Fluor 594 phalloidin (Cat# A12381; Thermo Fisher) and goat anti-rabbit, anti-mouse, or anti-rat Alexa Fluor-488 (Cat# A1101; Thermo Fisher) and 594 (Cat# A11037; Thermo Fisher) secondary antibodies. The chemical reagents used were as follows: 4’,6’-diamidino-2-phenykindole (DAPI) (Cat# 1331762; Bio-Rad Laboratories), Vectashield (Cat# H-1000; Vector Laboratories), PKH26 red fluorescent cell linker kit for general cell membrane labeling (Cat# PKH26GL-1KT, Sigma-Aldrich), PKH67 green fluorescent cell linker kit for general cell membrane labeling (Cat# PKH67GL-1KT, Sigma- Aldrich), CellTracker™ Deep Red Dye (Cat# C34565, Thermo Fisher), fibronectin human foreskin (Cat# F2518-5MG; Sigma-Aldrich), and type I collagen rat tail (Cat# MA01730; BD Biosciences).

### RNA isolation, RT-PCR, and qPCR

Total RNA was extracted from cells using Purezol Reagent (Cat# 7326880; Bio-Rad) according to the manufacturer’s instructions, and 1 μg was used for retrotranscription (RT) using the PrimeScript RT Reagent Kit (Cat# RR037A, Takara). cDNA was examined by semi-quantitative polymerase chain reaction (PCR), conducted in the QuantStudio™ 3 Real-Time PCR System (Applied Biosystems) using SensiFAST SYBR Hi-ROX mix (Cat# BIO-92020, Meridian). Final data were obtained by using 2^-ΔΔCt^ method. The primers used were as follows:

EDNRA primer 1: 5’-ATCACCGTCCTCAACCTCT-3’

EDNRA primer 2: 5’-CAGATGGAGAGACAATTTCAATGGC-3’

ARRB1 primer 1: 5’-CAGGAACGCCTCATCAAGA-3’

ARRB1 primer 2: 5’-GCAGTGTCACAGAACATGGA-3’

ITGB1 primer 1: 5’-GTAGCAAAGGAACAGCAGAGA-3’

ITGB1 primer 2: 5’-GGTCAATGGGATAGTCTTCAGC-3’

ITGA5 primer 1: 5’-ACCAACAAGAGAGCCAAAGTC-3’

ITGA5 primer 2: 5’-TTGTACACAGCCTCACACTG-3’

GAPDH primer 1: 5’-ACATCGCTCAGACACCATG-3’

GAPDH primer 2: 5’-TGTAGTTGAGGTCTGAAGGG-3’.

### Silencing, transient and stable transfections

Silencing of β-arr1 (L-011971-00) for 48 h [17] and talin1 (L-012949-00) for 72 h was performed using ON-TARGET plus SMART pool siRNAs, and siGENOME control pool non-targeting was used as a negative control (SCR) (Dharmacon). To silence Intβ1 (ITGB1), three selected pre-designed and validated siRNAs for a single-target gene were tested for their knockdown efficiency (hs.Ri.ITGB1.13.1, hs.Ri.ITGB1.13.2, and hs.Ri.ITGB1.13.3) (TriFECTa kit, IA, USA). The TriFECTa kit from IDT contained three specific dicer-substrate 27-mer RNA duplexes. Each TriFECTa kit contained a silencer negative control. In brief, 3 × 10^5^ cells were seeded and cultured in six-well plates until they reached 30–50% confluence and transiently transfected for 24 h, using lipofectamine RNAiMAX (Cat# 13778; Invitrogen) reagent according to the manufacturer’ silencing the cells were lysed to confirm efficient knockdown, total cell lysate was collected at the endpoint of each experiment and analyzed by western blotting. The best knockdown efficiency (75–90%) was obtained by employing hs.13.2 for Intβ1.

To obtain SKOV3-paxillin-GFP expressing cells, cells were plated at a density of 10,000/cm^2^ in a complete medium to reach 30–50% confluence at the time of infection. The medium was aspirated and changed to a culture medium containing viral particles at an MOI of 20, followed by incubation o/n. Cells were infected with GFP-paxillin lentiviral particles (Vector ID: VB190128-1060dng, VectorBuilder, Chicago, IL, USA) using polybrene (5 μg/mL, Santa Cruz Biotechnology). The following day, the cells were washed several times with PBS, and neomycin (G418) selection was performed at a concentration of 1 mg/ml for 7 days. After antibiotic selection, the culture medium was replaced with a complete growth medium.

### Western blotting (WB) and immunoprecipitation (IP)

For WB analysis, total cells were detached by scraping, collected by centrifugation, and lysed in RIPA buffer [50 mM Tris-HCl (pH 7.5), 150 mM NaCl, 1% Nonidet P-40, 0.5% sodium deoxycholate (NaDoc), 0.1% SDS], proteases (Cat# 3910201, SERVA), and phosphatase (Cat# 39055.02, SERVA) inhibitors. Protein concentrations were determined using the DC protein assay (Bio-Rad Laboratories). Cell lysates were resolved on MiniPROTEAN TGX gels and transferred to nitrocellulose membranes (Bio-Rad Laboratories), followed by incubation WB using primary antibodies, which were revealed using horseradish peroxidase-conjugated secondary antibodies. For immunoprecipitation (IP), precleared whole-cell lysates were incubated with specific antibodies or the corresponding IgG control (Thermo Fisher) and protein A or G Sepharose beads (GE Healthcare) at 4 °C overnight. For the detection of co-immunoprecipitated β-arr1, HRP-conjugated protein A peroxidase (Thermo Fisher) was used as a secondary antibody. Precision Plus Protein Standards Dual Color (Bio-Rad) was used as a molecular weight marker. Proteins were visualized by chemiluminescence (Clarity Western ECL Substrates, Bio-Rad Laboratories) by using Azure 300 (Azure Biosystems) and by Chemi Doc Imaging System and Image Lab Software (Biorad Laboratories). Quantification analyses were performed using ImageJ (https://imagej.net/software/fiji), a Java-based freeware, and reflected the relative amounts as a ratio of each protein band relative to the loading control of the lane.

### Immunofluorescence and confocal laser scanning microscopy (CLSM)

Cells cultured on coverslips were fixed with 4% paraformaldehyde for 10 min at room temperature, permeabilized with 0.2% Triton X-100, and blocked with 0.1 M glycine, 1% BSA, and 0.1% Tween20 in PBS for 30 min at room temperature. Samples were incubated with primary antibodies in 0.5% BSA in PBS overnight at 4 °C, followed by incubation with secondary antibodies for 1 h at room temperature. Coverslips were mounted using Vectashield mounting medium for fluorescence (Vector Laboratories). CLSM observations were performed with a Zeiss LSM980 confocal microscope, using a 63x/1.40 NA oil objective and excitation spectral laser lines at 405, 488, 543, 594, and 639 nm. Image acquisition and processing were carried out using Zeiss confocal software Zen 3.1 (Blue edition). Signals from different fluorescent probes were obtained in sequential scan settings, and colocalization was visualized in merged images.

For analysis of focal adhesion, CLSM observations were performed with a Nikon AR1 confocal microscope, using a 60×/1.40 NA oil objective and excitation spectral laser lines at 405, 488, 594, and 647 nm. Image acquisition and processing were performed using the Nikon NIS Elements Software and ImageJ (when applicable). Signals from different fluorescent probes were obtained in sequential scan settings, and colocalization was visualized in merged images. Several cells were analyzed for each labeling condition, and representative results are shown. The average focal adhesion density was calculated as the number of focal adhesions per cell normalized to the cell area. For fluorescence analysis, the cell cytoplasm was selected as the region of interest (ROI). Fluorescence intensity was calculated using ImageJ by dividing the mean fluorescent intensity over the area of the selected ROI/cell. All colocalization analyses were carried out using the Coloc2 plugin of ImageJ software to calculate Pearson’s correlation coefficients. This software estimates the degree of overlap between fluorescence signals obtained in two separate fluorescent channels. The Pearson’s coefficients were calculated from multiple images (*n* = 2–7) and then averaged and an SD of the mean was calculated.

### Proximity ligation assay (PLA)

Briefly, cells (1 × 10^4^) were cultured on slides in a 24-well plate, stimulated for 5 min, then fixed with 4% paraformaldehyde for 10 min at room temperature, washed in PBS, and blocked at 37 °C with Duolink blocking solution. After blocking, cells were incubated with the primary antibodies overnight at 4 °C. Cells were washed with PBS and then incubated with probe anti-mouse PLUS (Cat# DUO92001) and probe anti-rabbit MINUS (Cat# DUO92005; Sigma-Aldrich) for 1 h at 37 °C. Negative controls were obtained by incubating one primary antibody with irrelevant IgG. Coverslips were washed with PBS and then incubated with a DNA ligase diluted in ligation buffer for 30 min at 37 °C. After washing, coverslips were incubated with a DNA polymerase diluted in an amplification buffer for 120 min at 37 °C. The slides were then washed with PBS and incubated with DAPI for 10 min. Coverslips were mounted with Vectashield mounting medium for fluorescence. Red PLA signals (excitation/emission 598/634 nm) were identified as fluorescent spots using an Olympus AX70 microscope using a 40×/0.75 Ph2 objective. Images were acquired using a TCH-1.4ICE camera (Tucsen Photonics, China) controlled by the ISCapture. Images were imported into merged tiff formats containing both signal and nuclei channels. For each experimental condition, 15 randomly selected non-overlapping visual fields were analyzed and used for quantification analysis. DAPI images were used to mark the cell nuclei. For each image, foci were quantified using the “Find Maxima” tool in the FIJI software (noise tolerance = constant for the same set of images, output type = maxima within tolerance).

### Inverted 3D collagen invasion assay

In total, 100 μl of 2.0 mg/ml type I collagen/(15 μg/ml) fibronectin was allowed to polymerize in Transwell inserts (8-mm pores, Corning) for 2 h at 37 °C. Cells were seeded on top of the gel in a serum-free medium, and stimuli and/or inhibitors were added to the medium in the bottom chamber of the transwell as chemoattractants. After 48 h, the cells were fixed, stained, and visualized by CLSM using a Zeiss LSM980 apparatus, as described previously [17]. The invasion was measured using FIJI software by dividing the sum of the signal intensity of all slides beyond 20 μm (invading cells) by the sum of the intensity of all slides (total cells).

### Recombinant protein purification and GST-pulldown assay

The plasmid pGEX-4 T1 β-arrestin1 (a gift from Robert Lefkowitz, RRID: Addgene_36918A) was transformed into Escherichia coli BL21 by heat shock. Transformed cells were grown in LB medium, supplemented with 50 mg/ml ampicillin, till an OD_600_ of 0.6–0.7 and induced with 1 mM isopropyl-thiogalactoside (IPTG) for protein expression, at 25 °C overnight. The recombinant protein was purified from the cell pellet using the MagneGST Protein Purification System (Promega), according to the manufacturer’s instructions. For GST-pulldown assay, 300 μg of cell lysate, 10 μg of GST protein, and a GST-fusion probe were incubated together with 50 μl of Glutathione-Sepharose beads for 2 h at 4 °C with end-over-end mixing. The beads were immobilized on a magnetic stand and washed three times with PBS. After washing, the proteins were eluted in Laemmli 2× by heating to 95 °C for 5 min. Complexes recovered from the beads were analyzed by WB and GST-β-arr1 was detected using anti-GST Ab.

### Live–dead assay

Cells (1 × 10^3^) were cultured in complete medium in a U-shaped bottom 96-well plate and centrifuged at 200 g for 5 min. 24 h after spheroid formation, serum-free medium, as indicated, was added and left for 72 h at 37 °C. After 72 h, the Cyto3DTM Live–Dead Assay Kit (TheWell Bioscience, Inc., North Brunswick, NJ, United States) was used to determine the live/dead nucleated cells using a dual-fluorescence system of acridine orange (AO) and propidium iodide (PI), both nuclear staining (nucleic acid binding) dyes. All live nucleated cells fluoresce green, and all dead nucleated cells fluoresce red. Several images were obtained using a Bio-Rad ZOE fluorescent cell imager under a phase-contrast microscope (Bio-Rad Laboratories). Image analysis was performed using the FIJI software by calculating the mean gray value of the green and red channels separately, and then the green/red ratio was calculated.

### Cell adhesion assay

Cells (15 × 10^3^) after trypsinization were labeled with PKH67 green or PKH26 red fluorescent cell linker (Cat# MINI26-1KT; Sigma-Aldrich) for 5 min at 37 °C, washed twice, and added to a 96-well microplate, respectively, coated with fibronectin or a monolayer of human primary mesothelial cells on fibronectin. Cells were incubated with serum-free medium as indicated, and after 30 min, non-adherent cells were removed by washing three times with serum-free medium. Adherent cells were photographed using a Bio-Rad ZOE fluorescent cell imager (Bio-Rad Laboratories), and the results of the analysis of individual photos are reported. The number of adherent cells was quantified using the “Find Maxima” tool in FIJI software (noise tolerance = constant for the same set of images, output type = maxima within tolerance).

### Trans-mesothelial migration assay

Human primary mesothelial cells were seeded (1 × 10^5^) in 8.0-mm pore-sized membranes (Cat# 662638; Greiner Bio-one) coated with fibronectin (15 μg/ml) and left to form a monolayer for 48 h at 37 °C. Cells were stained with PKH26 cell linker (as reported above), washed with a complete medium, plated onto a mesothelial monolayer, and allowed to migrate for 12 h. Serum-free medium containing stimuli and inhibitors were then added to the lower chamber. Transmigrated cells were photographed using a Bio-Rad ZOE fluorescent cell imager (Bio-Rad Laboratories, RRID: SCR_008426), and the results of the analysis of the individual photos are reported. The number of migrating cells was quantified using the “Find Maxima” tool in FIJI software (noise tolerance = constant for the same set of images, output type = maxima within tolerance).

### Mesothelial clearance assay

OV.GEM#20 cell spheroids were formed by incubating 1 × 10^3^ cells per well in a 96-well U-bottom-shaped culture dish with a cell-repelling surface (Cat# F202003, faCellitate) at 37 °C for 16 h. Cell-repelling surface prevented the cells from attaching to the culture dish, allowing them to remain in suspension and form a single cluster per well. MCs were labeled with 2 × 10^−6^ M PKH26 red fluorescent cell linker for 5 min, and washed two times. Then, MC monolayer was prepared by plating 40 × 10^3^ cells per well in fibronectin-coated (50 μg/ml) Ibidi chamber slides (μ-slide 18 well Uncoated, Ibidi, Cat# 81811) followed by incubation at 37 °C for 16 h. The spheroids were then transferred to the slides with the MC monolayers and the two cell populations were imaged. For live imaging experiments, we used an X-Light V3 confocal spinning disk unit (CrestOptics) mounted on a Nikon Ti-E Inverted Motorized time-lapse microscope with integrated Perfect Focus System and Differential Interference Contrast (DIC) optics and equipped with a Kinetix CMOS camera (Photometrics) and a Celesta laser source (Lumencor). Ibidi slides were placed in the time-lapse microscope incubation chamber with integrated temperature, CO_2,_ and humidity control (OkoLab), and time-lapse Z-stack acquisitions were conducted for 24 h at 30 min intervals using NIS Elements AR ver.5 software (Nikon). Images represent the Maximum Intensity Projection (MIP) of 40 µm Z-stacks (z.step 0.9 µm).

SKOV3 cell spheroids were formed by incubating 1 × 10^3^ cells per well in a 96-well U-bottom-shaped culture dish with a cell-repelling surface (Cat# F202003, faCellitate) at 37 °C for 16 h. MC monolayer is prepared by plating 40 × 10^3^ cells per well (pre-stained for 30 min with 0.75 μM CellTracker Deep Red) in fibronectin-coated (50 μg/ml) 96-well microplate and incubating the plate at 37 °C for 16 h. The spheroids were then transferred to the dish with the MC monolayer and the two cell populations were imaged. Spheroid-induced mesothelial clearance was monitored by time-lapse microscopy using an epifluorescence inverted microscope (Nikon Eclipse Ti-E) equipped with an Okolab cage incubator for temperature and CO_2_ control. Fluorescence and phase-contrast images (×20 objective) were collected for each experimental condition for 24 h at 30 min intervals. For each time point, the non-fluorescent area in the mesothelial monolayer underneath the spheroid was measured by NIS Elements AR software (Nikon) and normalized to the initial spheroid area. Experiments were conducted at least in triplicate.

### 3D organotypic cultures

The organotypic model was created by putting type I collagen (0.8 mg/ml)/fibronectin (50 μg/ml) and a monolayer of mesothelial cells (5 × 10^5^) on the Alvetex scaffolds (Reinnervate, Sedgefield, Co. Durham, UK). Once mesothelial cells formed a monolayer, tumor cells (2 × 10^6^) were seeded and cultured for 7 days. After 2 days, the growth medium was replaced with a starved medium under the conditions indicated. After 7 days, the scaffolds, once the medium is removed, are washed with PBS, fixed with Bouin’s solution for 16 h, dehydrated with sequential ethanol washes (30–95%), clarified in xylene, and sliced into two parts and embedded on Formalin Fixed Paraffin Embedded (FFPE). The scaffolds were sectioned at 10 μm, deparaffinized with xylene and ethanol, hydrated through graded alcohols, and subjected to heat-induced epitope retrieval step by pH6 citrate buffer (Novus Biologicals) three times for 3 min in a microwave. Sections were washed with PBS-T (0.01% Tween20) and blocked in PBS-BSA 3% for 30 min at 37 °C, and then stained in two different ways: (1) with the standard hematoxylin (Mayer’s solution for hematoxylin Sigma-Aldrich, Cat# MHS16) and eosin (alcoholic Eosin Y solution, Sigma-Aldrich, Cat# HT110116l) technique; (2) for active Intβ1 detection (Cat#550531; BD Biosciences) was added to PBS-BSA (0.5% BSA) and incubated 30 min at 37 °C, followed by incubation with Alexa Fluor-488 F(ab)2 fragments of goat anti-rat IgG plus DAPI (Thermo Fisher Scientific). The slides were mounted in Vectashield (Vector Laboratories) and observed on a Zeiss LSM980 confocal laser scanning microscope.

### In vivo experiments

For in vivo animal studies, the experimental protocols complied with the principles of ARRIVE (https://arriveguidelines.org) and were approved by the National Ethics Committee for Animal Experimentation of the Italian Ministry of Health (authorization N1/2020- PR #365869604). The mice were housed in single cages with wood-derived bedding material in a specific pathogen-free facility with a 12 h light/dark cycle under controlled temperatures. The mice were cared for under the principles of laboratory animal care (National, Bethesda, Dof USA no. 85–23, revised 1985) and national laws, and received water and food ad libitum. 4–6 weeks of age female NOD/SCID mice (Charles River Laboratories) were used. Mouse in vivo adhesion assays were performed as follows: 2 × 10^6^ viable SKOV3-Luc cells were injected into the peritoneal cavity (i.p.) of mice (*n* = 5 per group) [19]. After 24 h, the mice were sacrificed. The peritoneum, omentum, and mesentery were excised and ex vivo analysis was performed. After gentle washing with PBS to eliminate non-adherent cells, the luminescence of adherent cell aggregates was read and recorded. To test the antitumor metastatic efficacy of AMB, mice were injected i.p. with 200 μL PBS containing 2 × 10^6^ viable SKOV3-Luc cells following the guidelines for animal experimentation. Two weeks later, animals were randomized into four different groups and subjected to the following treatments for 5 weeks: (i) 200 μL Methocell (vehicle control, CTR), (ii) 200 μL AMB (10 mg/kg/mice, daily, oral gavage), (iii) 200 μl ATN161 (100 mg/kg mice, twice a week, i.p.), and (iv) AMB and ATN161 in combination. The experiment was performed twice, with five mice per treatment arm per experiment. Mice were observed two times per week and monitored for signs of distress (i.e., changes in appearance, respiration, activity, etc.) and weighed. Mice showing signs of distress or losing greater than 15% body weight were euthanized. Tumor burden was assessed on days 17, 25, 32, and 39 after tumor cell injection by measuring the light emission following i.p. luciferin administration (no blinding was done). Briefly, 10 min after administration of D-luciferin (75 mg/kg body weight, intraperitoneal; Perkin Elmer, Hopkinton, MA, USA), photon emission was acquired for 5 min and analyzed using a CCD camera (Xenogen IVIS Lumina System; Perkin Elmer). The total flux (photons/s) was determined for the entire abdominal cavity per mouse and normalized to the mean total flux of the control-treated mice imaged on day 17. Upon experimental termination (day 39), the mice were euthanized, and the number of visible metastases was measured, carefully dissected, frozen, or embedded on paraffin, and analyzed by WB blotting and CLSM analysis.

### Bioinformatics analysis

The correlation of the combination of EDNRA (204464_s_at) and ITGB1 (211945_s_at) (mRNA expression to overall survival (OS) and progression-free survival (PFS) in ovarian cancer patients with serous ovarian cancer (all stages, grade 2 + 3) from all datasets was analyzed using the Kaplan–Meier plotter web tool (http://kmplot.com/analysis/index.php?p=service&cancer=ovar) [50]. Ovarian cancer patients were followed up for 60 months. To determine prognostic value, the samples were split into two groups according to the mean expression of the selected probes (user-selected probe set). mRNA expression above or below the median separates the cases into high- and low-expression levels. Hazard ratios (HR), 95% confidence intervals, and log-rank P are presented in the main plots. The cBioPortal database analysis cBioPortal (The cBio Cancer Genomics Portal) database is a publicly accessible online database (http://www.cbioportal.org/; RRID:SCR_014555) [51, 52], which provides visualization and analysis tools for more than 715 datasets and 86,733 samples. The term “ITGB1” and “EDNRA” were used to search the cBioPortal database and the Ovarian Serous Cystadenocarcinoma (TCGA, Firehose Legacy), *n* = 617) cohort was used. All gene correlations were performed using the “co-expression” feature, and RNA Seq V2 RSEM data were used.

### Statistical analysis

Statistical analysis was conducted using GraphPad Prism software (https://www.graphpad.com/scientific-software/prism), and the values are presented as the mean ± SD with three independent experiments with similar results, otherwise indicated. We used cell cultures with a normal distribution and similar variance between groups. Graphs comparing the two conditions were analyzed using a two-sided unpaired *t* test. Graphs comparing more than two conditions were analyzed using one-way analysis of variance (ANOVA) followed by Tukey’s correction for multiple comparisons. Statistical significance was defined as *P < 0.05; **P < 0.01; ***P < 0.001; ****P < 0.0001. ns not significant.

### Reporting summary

Further information on experimental design is available in the Nature Research Reporting Summary linked to this article.

## Supplementary information

Supplementary TABLE1

Supplementary Figures

Supplementary video S1-CTR

Supplementary video S2-ET-1

Supplementary video S3-AMB

Supplementary video S4-AMB+ET-1

Supplementary video S5-ATN161

Supplementary video S6-ATN161+ET-1

Supplementary video S7- AMB+ATN161

Supplementary video S8- AMB+ATN 161+ET-1

Supplementary video S9-Y27632

Supplementary video S10-Y27632+ET-1

Original Data File

Reporting summary

## Data Availability

Data generated during this study are included in this article and its supplementary information file and could be available on reasonable request by inquiring the corresponding author. Uncropped western blots can be seen in supplemental materials.
