# Author Correction: The interaction of β-arrestin1 with talin1 driven by endothelin A receptor as a feature of α5β1 integrin activation in high-grade serous ovarian cancer

**DOI:** 10.1038/s41419-024-07139-x

**Published:** 2024-10-21

**Authors:** Ilenia Masi, Flavia Ottavi, Danila Del Rio, Valentina Caprara, Cristina Vastarelli, Sara Maria Giannitelli, Giulia Fianco, Pamela Mozetic, Marianna Buttarelli, Gabriella Ferrandina, Giovanni Scambia, Daniela Gallo, Alberto Rainer, Anna Bagnato, Francesca Spadaro, Laura Rosanò

**Affiliations:** 1 Institute of Molecular Biology and Pathology, CNR, Rome, 00185 Italy; 2 Unit of Preclinical Models and New Therapeutic Agents, IRCCS—Regina Elena National Cancer Institute, Rome, 00144 Italy; 3 Department of Engineering, Università Campus Bio-Medico di Roma, via Álvaro del Portillo 21, Rome, 00128 Italy; 4 Institute of Nanotechnology (NANOTEC), National Research Council (CNR), c/o Campus Ecotekne, via Monteroni, Lecce, 73100 Italy; 5 San Raffaele Hospital, Division of Neuroscience, Institute of Experimental Neurology, San Raffaele Scientific Institute, Via Olgettina, 60, Milan, 20132 Italy; 6 Dipartimento Universitario Scienze della Vita e Sanità Pubblica-Sezione di Ginecologia ed Ostetricia—Università Cattolica del Sacro Cuore, Largo A. Gemelli 8, Rome, 00168 Italy; 7 Dipartimento Scienze della Salute della Donna, del Bambino e di Sanità Pubblica, Fondazione Policlinico Universitario A. Gemelli, IRCCS, Largo A. Gemelli 8, Rome, 00168 Italy; 8 Confocal Microscopy Unit, Core Facilities, Istituto Superiore di Sanità, Rome, 00161 Italy

**Keywords:** Ovarian cancer, Cancer, Cell biology

Correction to: *Cell Death and Disease* 10.1038/s41419-023-05612-7, published online 30 January 2023

The authors regret that there is a mistake in Fig. 8B as published in the original article. In the first published version of this manuscript, the bioluminescent image of intraperitoneally (i.p) SKOV3-Luc-injected mice for AMB group was accidentally misused during the assembly of the figures. We greatly apologize for this error and are now providing a corrected version of the figure (see new Fig. 8B). The scientific conclusions of our study are not affected by this inadvertent error.

New figure 8
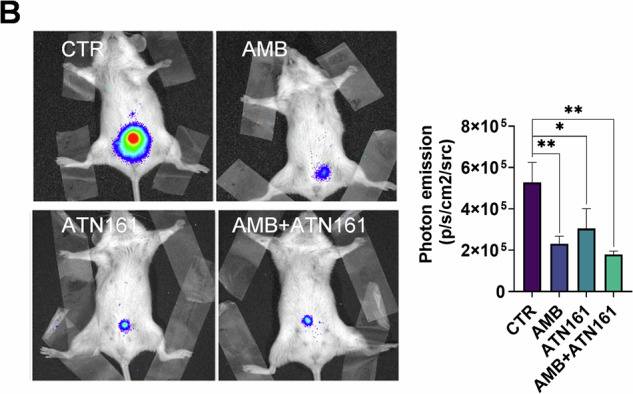


Old figure 8
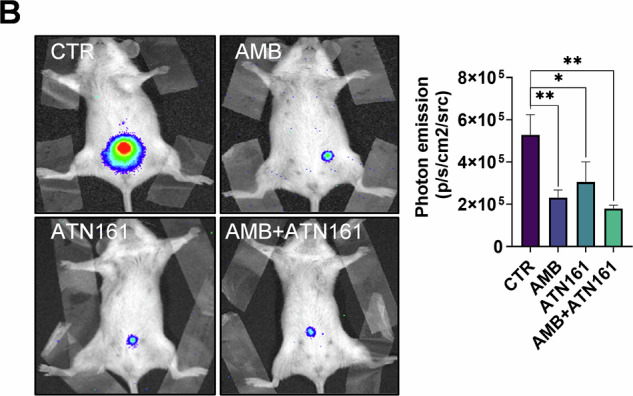


The original article has been corrected.

