# Supplementary material for: The interaction of β-arrestin1 with talin1 driven by endothelin A receptor as a feature of α5β1 integrin activation in high-grade serous ovarian cancer
Source: Cell Death Dis. 2023 Jan 30;14(1):73. doi: 10.1038/s41419-023-05612-7 (PMC9886921; doi:10.1038/s41419-023-05612-7)
Supplement: Supplementary file 1 — Supplementary TABLE1 [file 41419_2023_5612_MOESM1_ESM.docx]

**TABLE S1.** Clinicopathological features of HGSOC patients whose specimens were used for primary cell line isolation

| **Primary cell line** | **Age** | **Primary site** | **Figo Stage** | **Somatic BRCA** | **P53** | **CK7** | **PAX8** | **WT1** | |
| --- | --- | --- | --- | --- | --- | --- | --- | --- | --- |
| **OV.GEM-9** | 38 | Ovary | IVB | WT | Mut | *na* | *na* | *na* |  |
| **OV.GEM-11** | 65 | Ovary | IIIC | WT | Mut | + | + | + |  |
| **OV.GEM-20** | 62 | Peritoneum | IIIC | WT | Mut | + | + | + |  |
| **OV.GEM-27** | 42 | Omentum | IIIC | WT | Null | *na* | + | + |  |

HGSOC=high-grade serous ovarian cancer; *na*= not available
