# Supplementary material for: The interaction of β-arrestin1 with talin1 driven by endothelin A receptor as a feature of α5β1 integrin activation in high-grade serous ovarian cancer
Source: Cell Death Dis. 2023 Jan 30;14(1):73. doi: 10.1038/s41419-023-05612-7 (PMC9886921; doi:10.1038/s41419-023-05612-7)
Supplement: Supplementary file 2 — Supplementary Figures [file 41419_2023_5612_MOESM2_ESM.pdf]

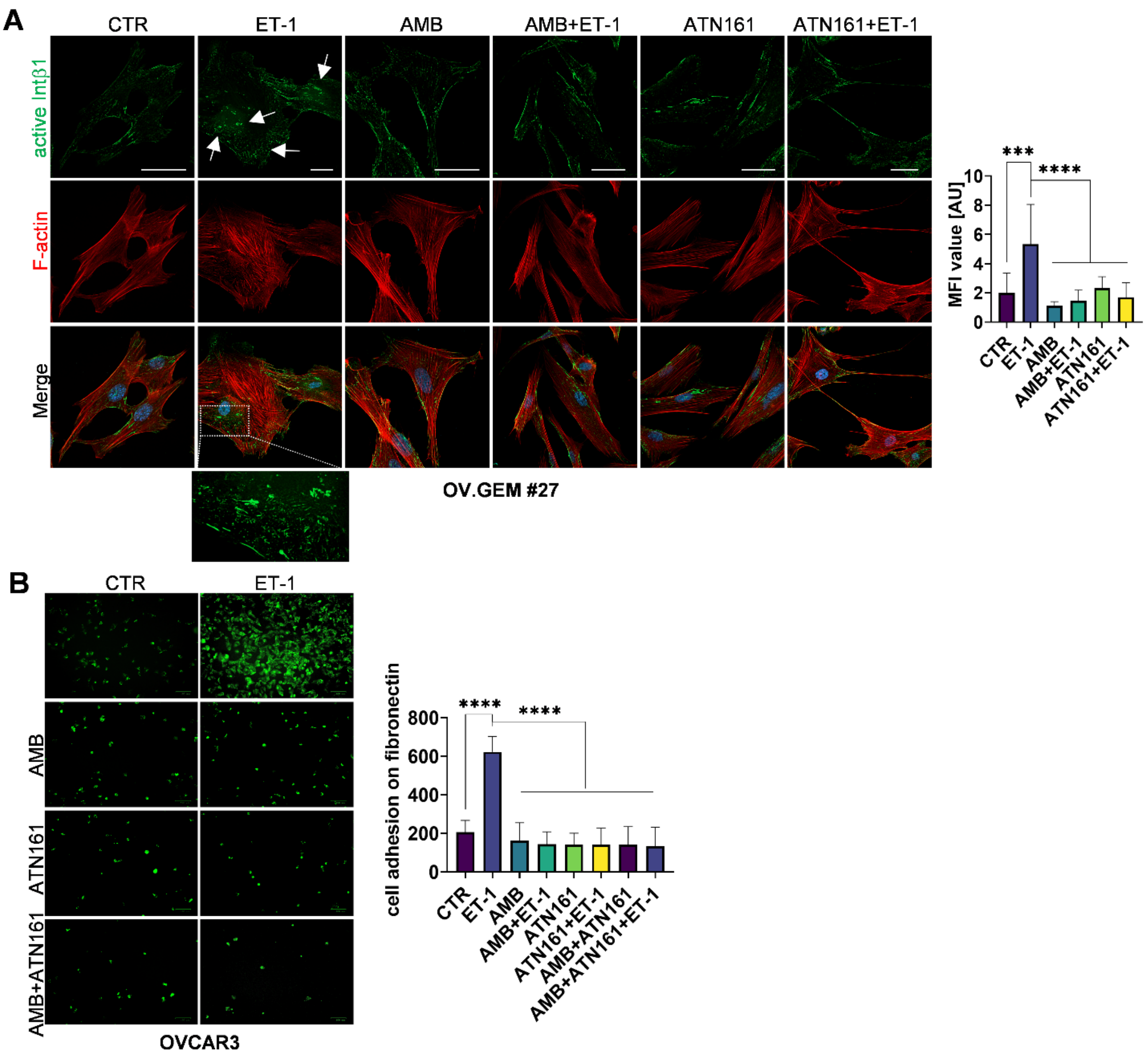

Supplementary Figure 1. ET-1/ET$_A$R activates Intβ1 signaling. (A) CLSM analysis cells stimulated with ET-1 (100nM) for 5 min and/or AMB (1 μm) and/or ATN161 (1 μm), stained for active Intβ1 (green) and F-actin (red). Nuclei are reported in blue (DAPI). Scale bar, 20 μm. For active Intβ1 a higher-power magnification image of a selected ROI in ET-1 stimulated cells is shown, indicating active Intβ1 intracellular accumulation. Scale bar, 50 μm. Histograms, mean fluorescence intensity (MFI) of active Intβ1/cytoplasmic area means ±SD. n=2, One-way ANOVA. **(B)** Cells were seeded on fibronectin (15 μm/ml) after labeling with PKH67 and stimulated with ET-1 and/or AMB and/or ATN161 for 30 min. Images of adherent cells were captured. Histograms, means ± SD from the adherent cells in 15 different fields. n=3, One-way ANOVA, Tukey post hoc analysis.

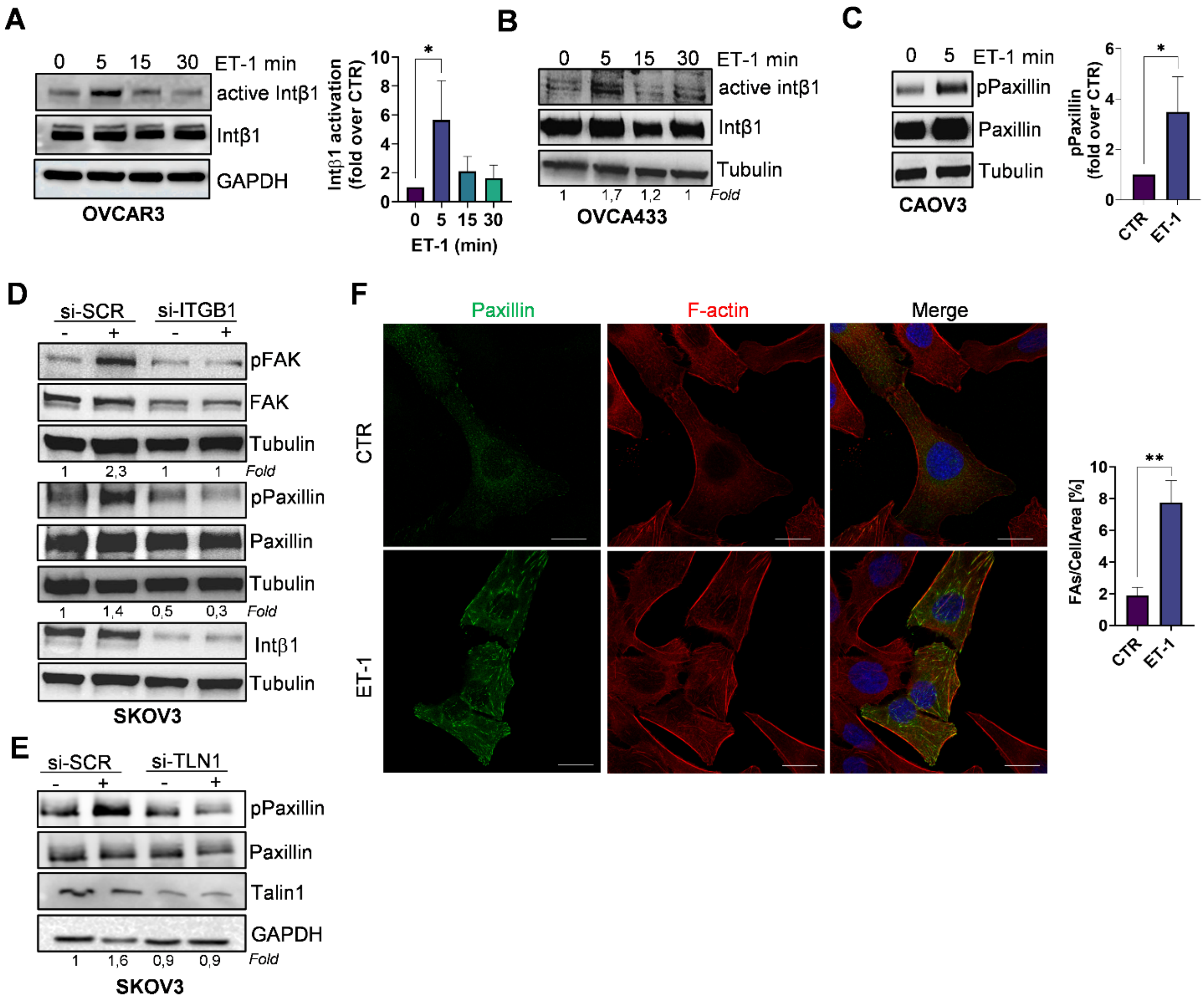

Supplementary figure 2. ET-1/ET_AR/β-arr1 activates Intβ1 and downstream signaling. (A), (B) and **(C)** Lysates of cells stimulated with ET-1 for indicated times were subjected to WB for indicated proteins. GAPDH and tubulin were used as loading control. Histograms, means ±SD of the average band intensity normalized to Tubulin or GAPDH (fold changes versus CTR) used as loading control; One-way ANOVA (A) and t-test (C). **(D)** si-SCR and si-ITGB1 transfected cells, stimulated with ET-1 for 5 min, were subjected to WB for indicated proteins. Tubulin was used as a loading control. **(E)** si-SCR and si-TLN1 transfected cells, stimulated with ET-1 for 5 min, were subjected to WB for indicated proteins. GAPDH was used as a loading control. **(F)** CLSM analysis of SKOV3 cells stimulated with ET-1 for 5 min and stained for paxillin (green) and F-actin (red) detection. Co-localization is shown in merged images. Nuclei are reported in blue (DAPI). Scale bar, 10 μm. Histograms, focal adhesion (FA)/cell area (%).

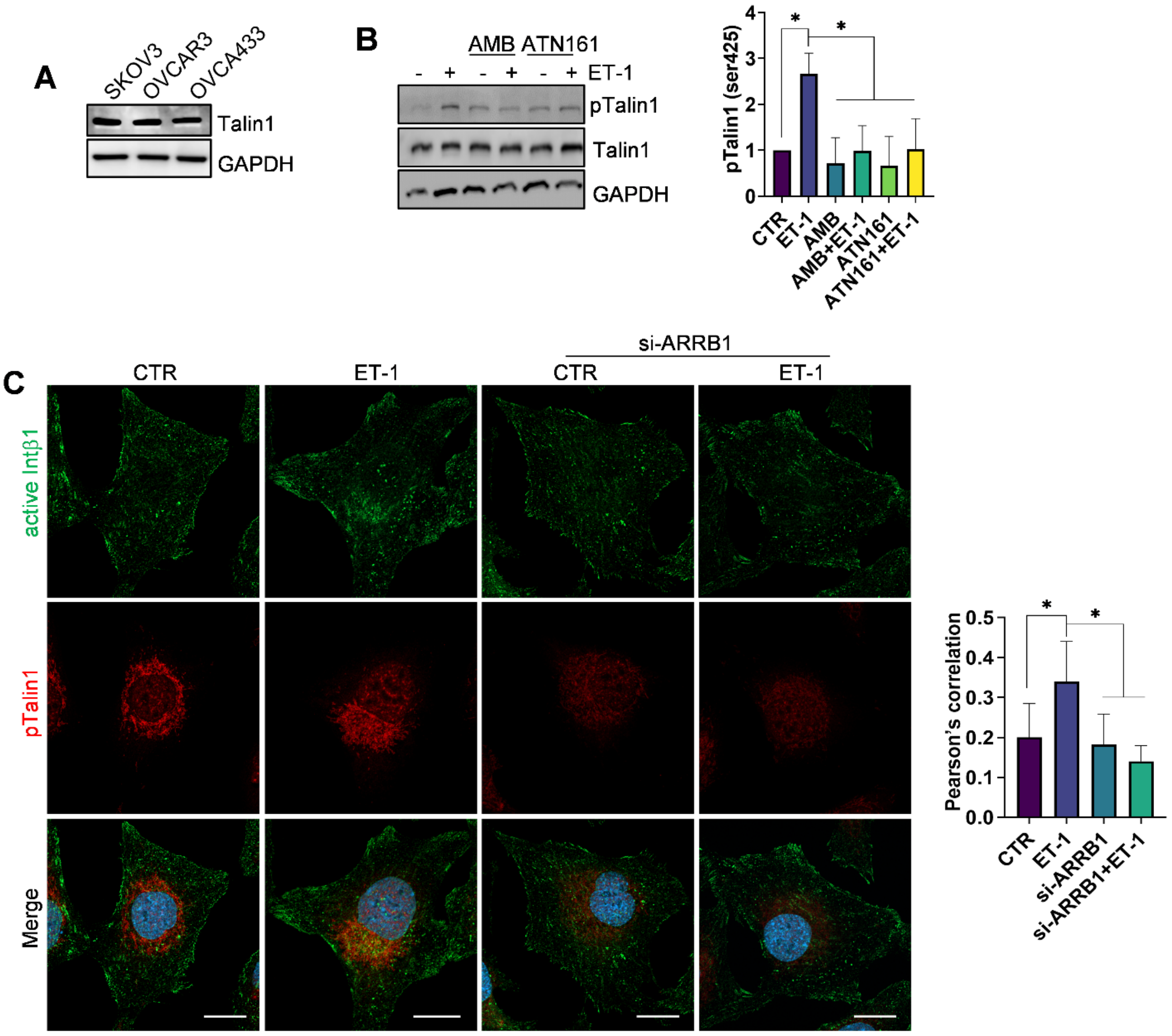

Supplementary Figure 3. ET-1 promotes talin1 phosphorylation and its association with active Intβ1. (A) WB analysis of talin1 expression in indicated cell lines. **(B)** Lysates of OVCA433 cells stimulated with ET-1 for 5 min and/or AMB and/or ATN161 were subjected to WB for indicated proteins. GAPDH was used as a loading control. Histograms, means ± SD of the average band intensity normalized to GAPDH (fold changes versus CTR); n = 3, One-way ANOVA. **(C)** CLSM analysis of SKOV3 cells transfected with SCR or si-ARRB1, stimulated with ET-1 for 5 min and stained for active Intβ1 (green) and ptalin1 (red). Co-localization is shown in merged images, detected in yellow. Nuclei are reported in blue (DAPI). Scale bar, 20 µm. Columns show the mean ±SD of quantification of Pearson's correlation between active Intβ1 and ptalin1. n = 2, One-way ANOVA

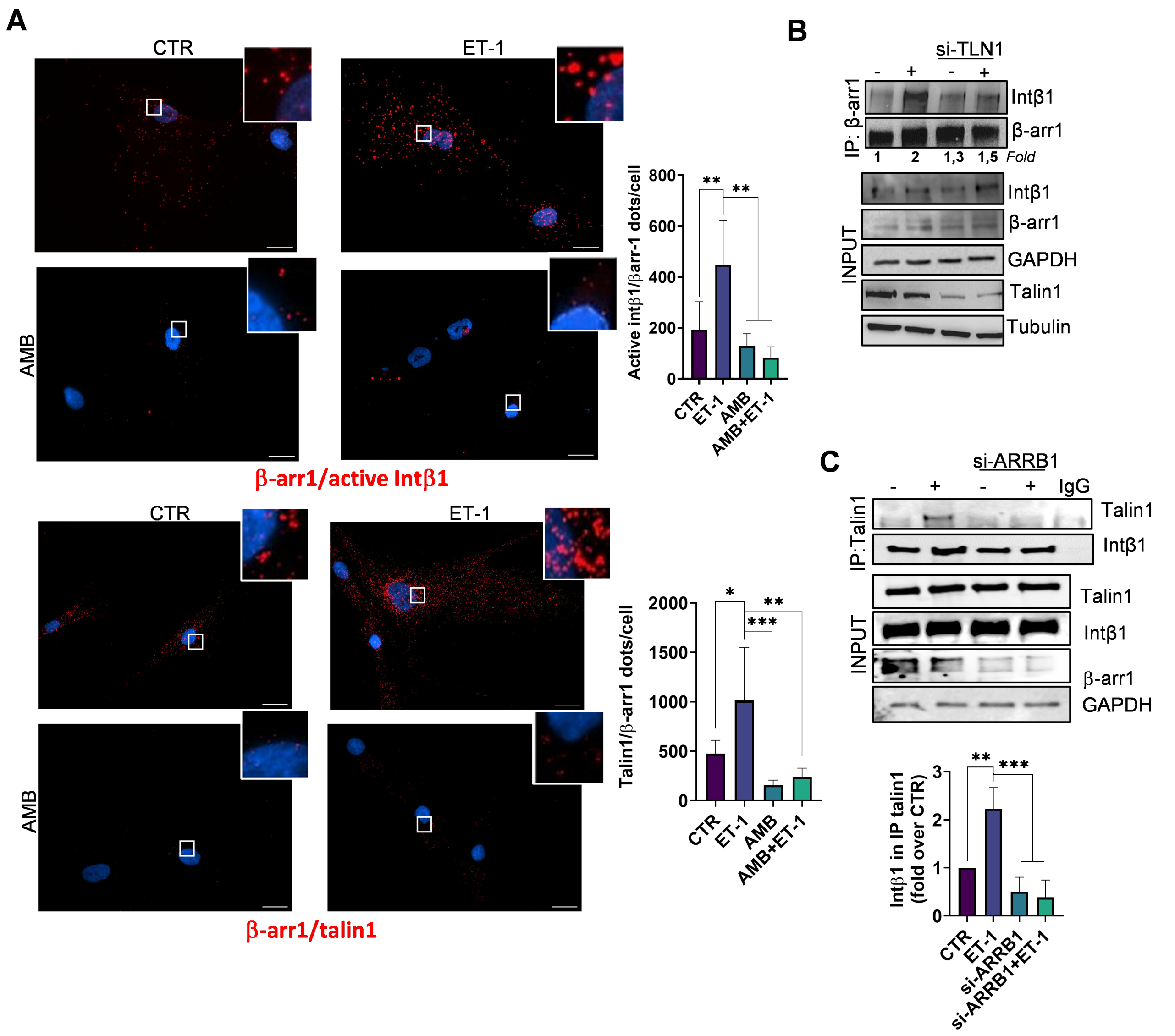

Supplementary figure 4. β-arr1 links Intβ1 and talin1. (A) Representative PLA images of protein complexes containing β-arr1 and Intβ1 or Talin1 in OV.GEM#20 cells stimulated with ET-1 and/or AMB for 60 min. The red signal represents a positive PLA reaction and DAPI staining (blue) highlights the nucleus. Scale bar, 10 µm. Inset, show higher magnifications of the square. Histograms, means ± SD of PLA dots per nucleus; n = 2. One-way ANOVA, Tukey post hoc analysis. **(B)** si-SCR and si-TLN1 transfected SKOV3 cells stimulated with ET-1 for 5 min, were IP with anti-β-arr1. IPs and inputs were subjected to WB for indicated proteins. The fold change of Intβ1 versus CTR in indicated protein IP normalized to INPUT is reported. **(C)** si-SCR and si-ARRB1 transfected SKOV3 cells stimulated with ET-1 for 5 min were IP with anti-talin1 or irrelevant IgG. IPs and inputs were subjected to WB for indicated proteins. Histograms indicate means ± SD of the average band intensity of Intβ1 in talin1 IP normalized to inputs (fold changes versus CTR). n = 3, one-way ANOVA, Tukey post hoc analysis.

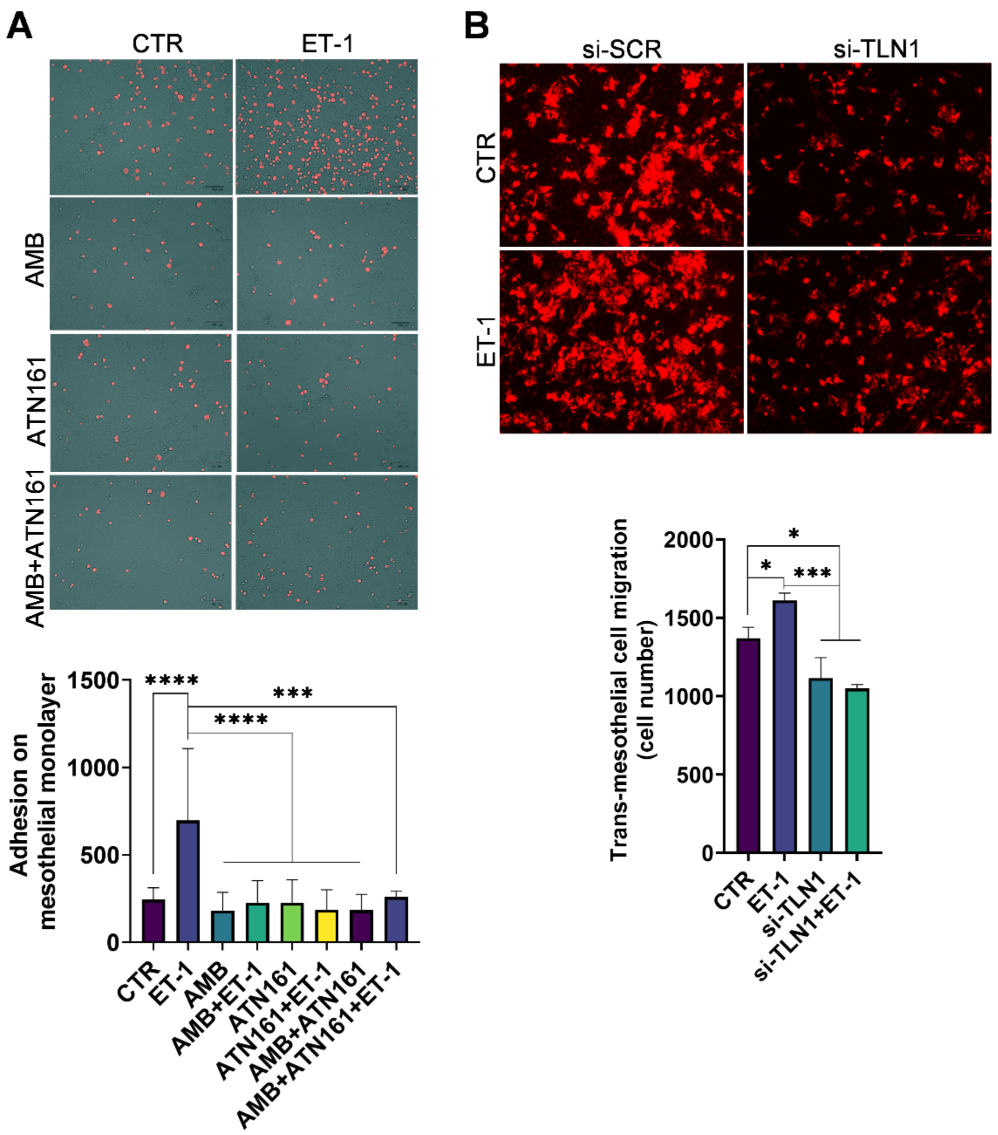

Supplementary figure 5. SOC cell adhesion and mesothelial clearance are regulated by ET-1/Intβ1 signaling. (A) SKOV3 cells were seeded into a monolayer of mesothelial cells on fibronectin (15 μm/ml) after labeling with PKH26 and stimulated with ET-1 and/or AMB and/or ATN161 for 30 min. Images of adherent cells were captured. Histograms, means ± SD from the quantification signaling in 15 different fields. n=3, One-way ANOVA, Tukey post hoc analysis. **(B)** SKOV3 cells (red), transfected with si-SCR and si-TLN1 were applied into the upper chamber with a monolayer of mesothelial cells on a fibronectin-coated membrane and stimulated with ET-1. Cells migrated through the mesothelial layer, and fibronectin and membrane pores were photographed. Histograms mean ± SD from the quantification of signals in 9 different fields. One-way ANOVA, Tukey post hoc analysis. Scale bar, 100 μm.

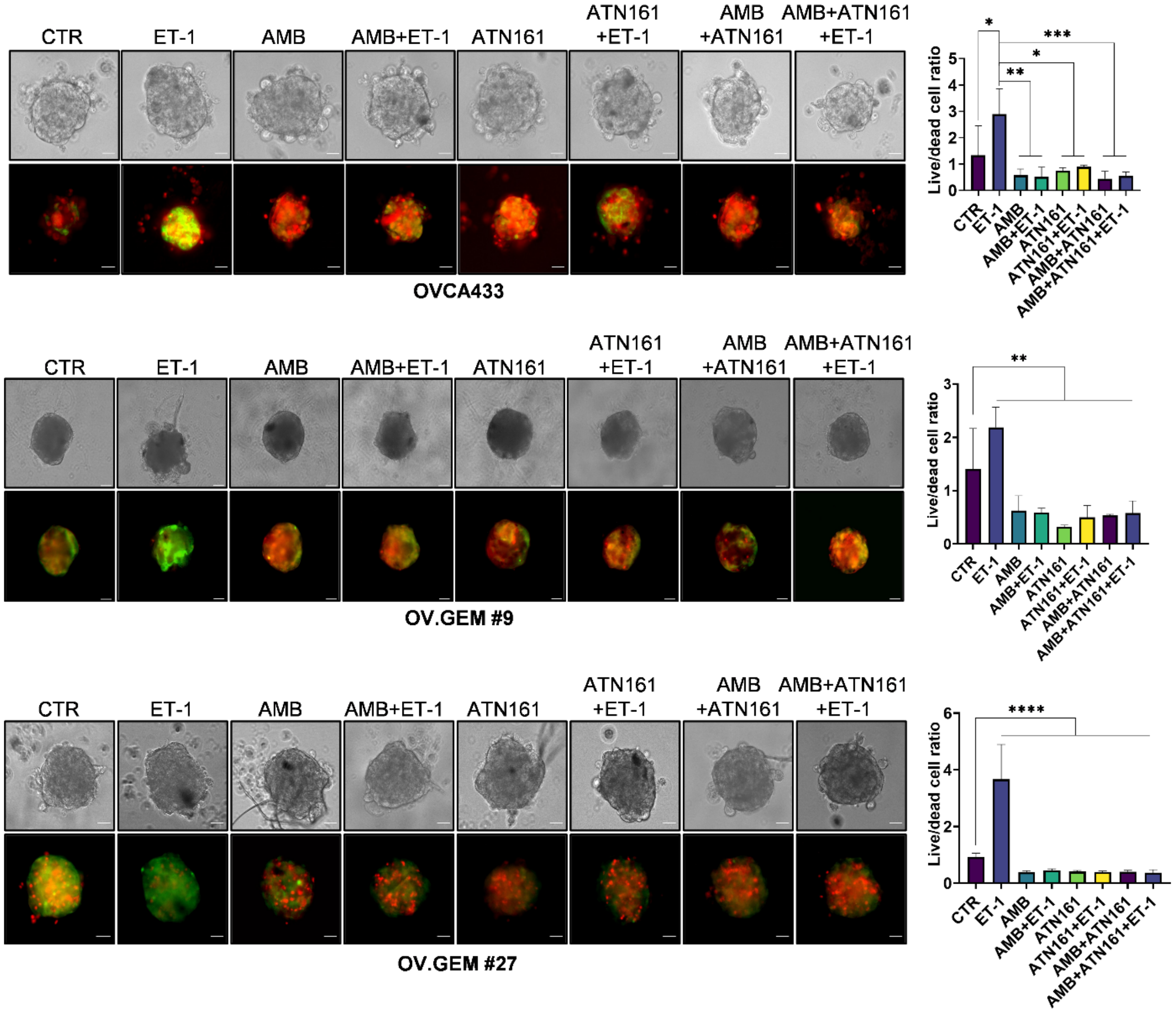

Supplementary figure 6. HG-SOC cell spheroid survival is regulated by ET-1/Intβ1 signaling. 3D spheroids were treated with ET-1 and/or AMB and/or ATN161 for 72 hr and live (green) or dead (red) cells were determined using a dual fluorescence system. Histograms, means ± SD of the live/dead cell ratio (fold changes versus CTR); n = 3 (OVCA433), n=2 (OV.GEM), One-way ANOVA.

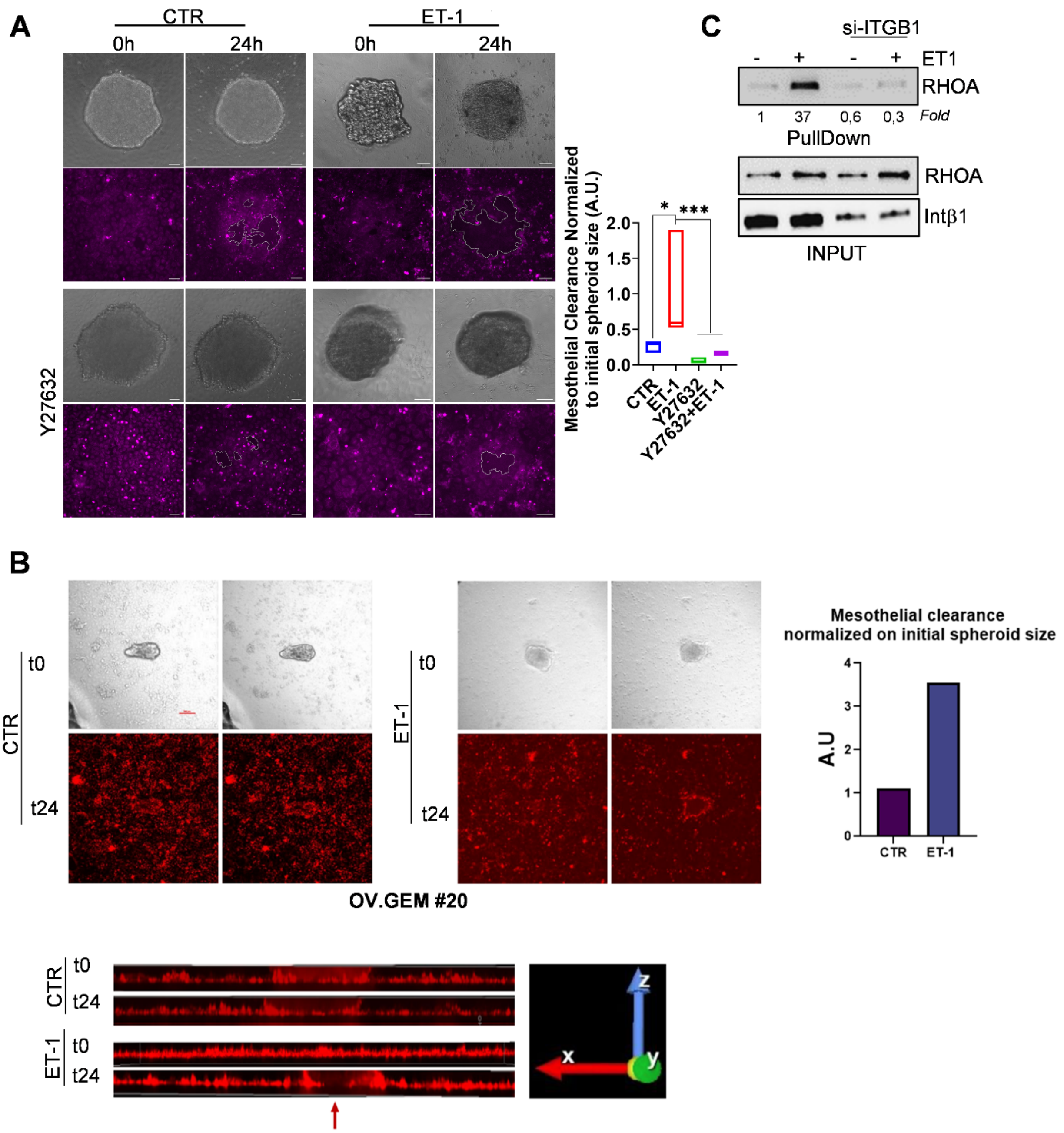

Supplementary figure 7. HG-SOC cell spheroid mesothelial clearance is regulated by ET-1 signaling. (A) Images depict mesothelial clearance induced by SKOV3 spheroids treated with ET-1 and/or Y2732 at 0- and 24-hr time points. Scale bar, 50 µm. The graph represents the ratio between the area of the "hole"/aperture in the mesothelial monolayer after 24h (highlighted with the white line) and the initial spheroid area (0h). n=2, one-way ANOVA. **(B)** Human primary HG-SOC cells (OV.GEM #20) on top of a confluent monolayer of a monolayer of mesothelial cells (red) and treated with ET-1. The dynamics of these two cell populations were followed in parallel for 24 hr. Images show a time course of mesothelial clearance at the initial time (t0) and after 24 hr (t24). Time-lapse images of multiple Z sections (side view) of OV.GEM #20 spheroids inducing clearance. Scale bar = 100µm. The graph represents the ratio between spheroid t24 area and t0 area on the mesothelial cell monolayer. The measurements were performed by ImageJ Fiji. **(C)** Rhotekin beads were used to pull down RhoA-GTP from si-SCR and si-ITGB1 transfected SKOV3 cells stimulated with ET-1 for 5 min. Pull-down samples and inputs were analyzed by WB for the indicated proteins.
