# Supplementary material for: The interaction of β-arrestin1 with talin1 driven by endothelin A receptor as a feature of α5β1 integrin activation in high-grade serous ovarian cancer
Source: Cell Death Dis. 2023 Jan 30;14(1):73. doi: 10.1038/s41419-023-05612-7 (PMC9886921; doi:10.1038/s41419-023-05612-7)

Figure 2B

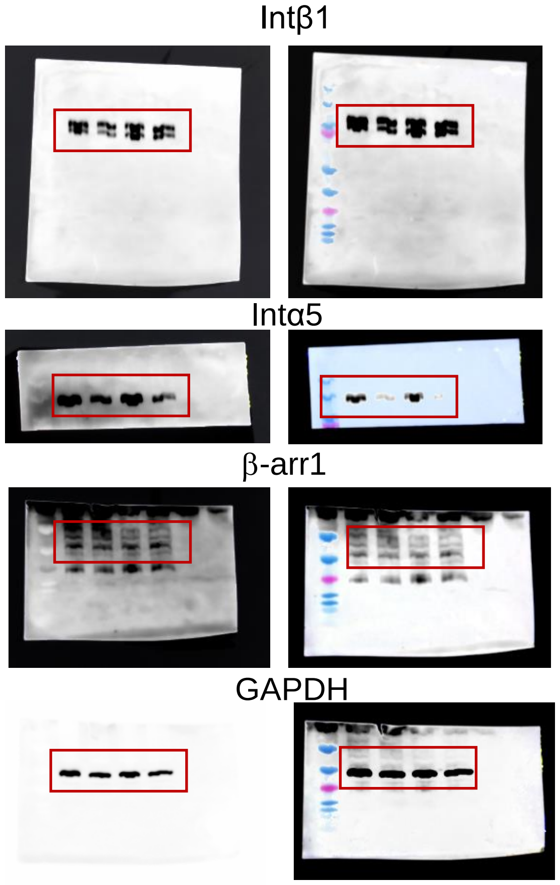

Figure 2C

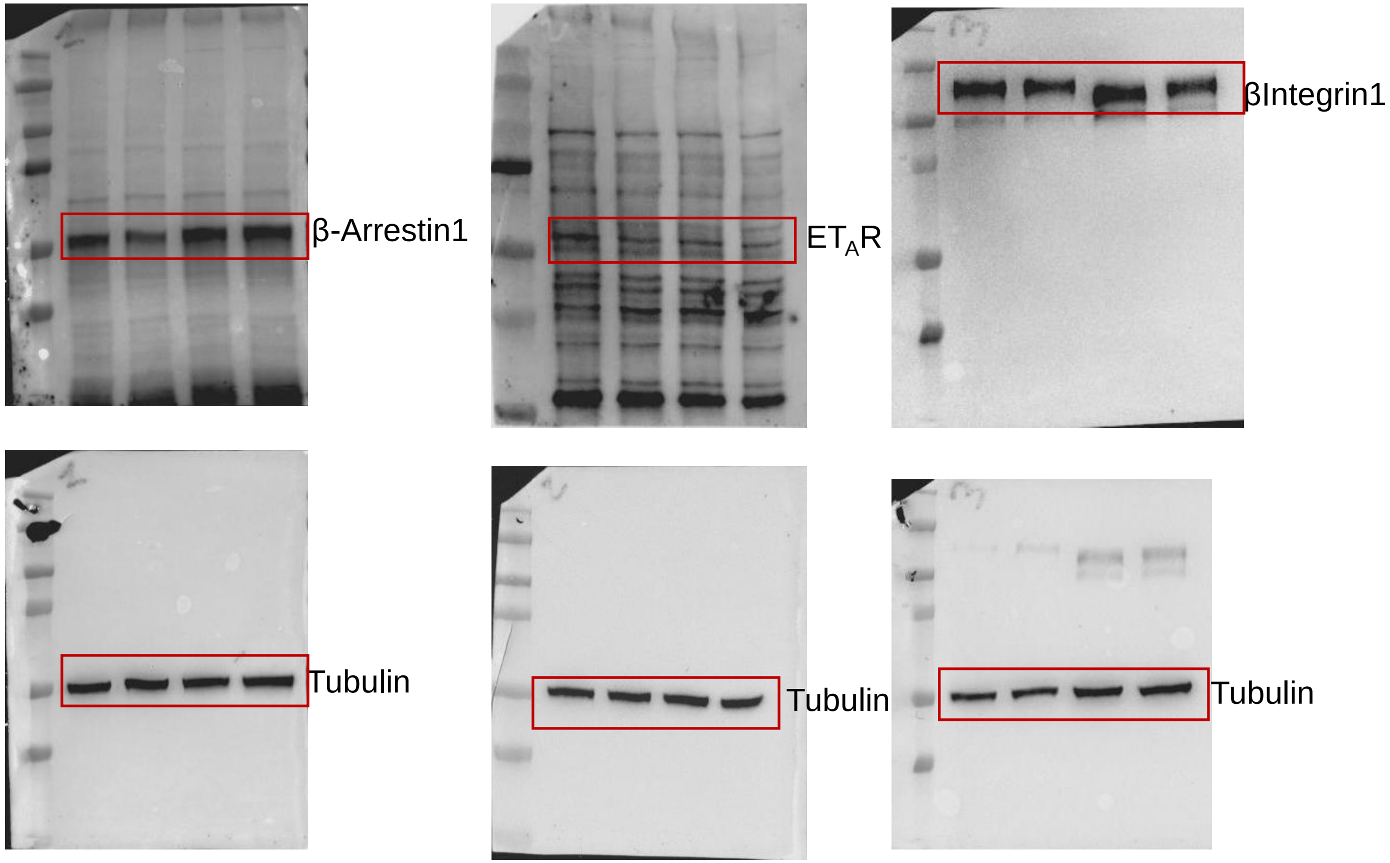

Figure3B

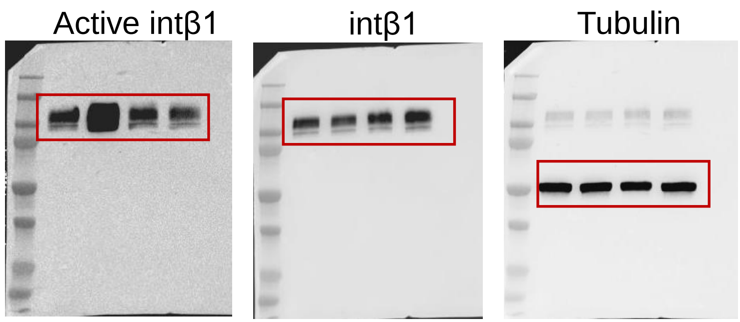

Figure3C

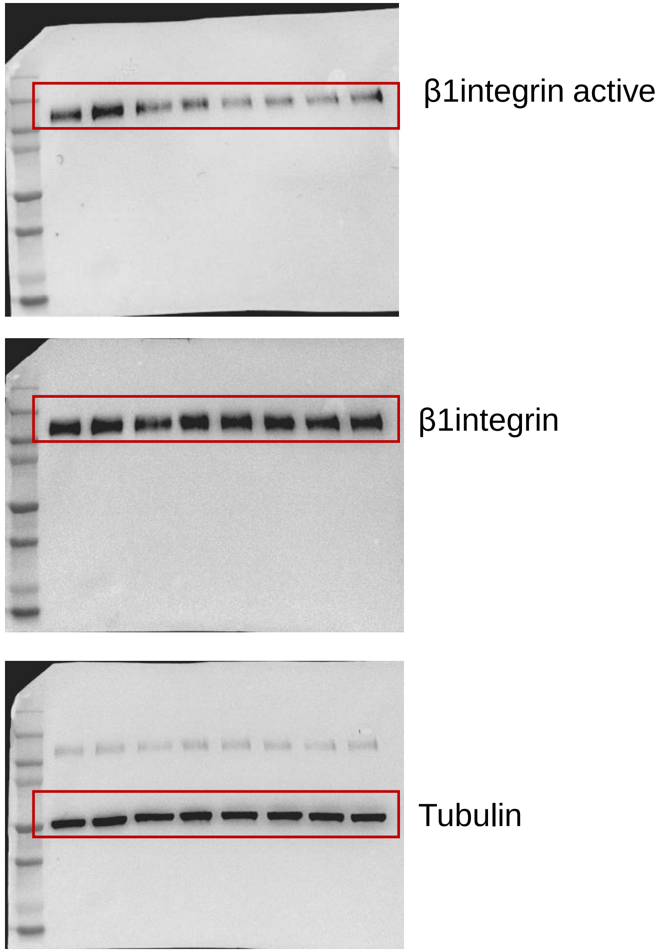

Figure 5A

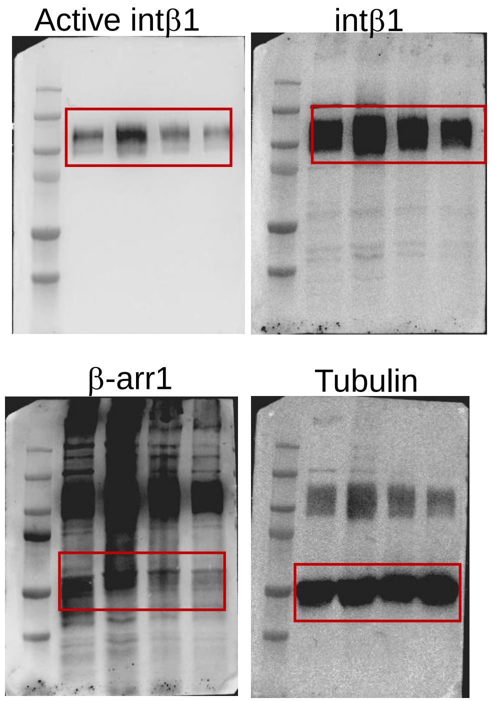

Figure 5B

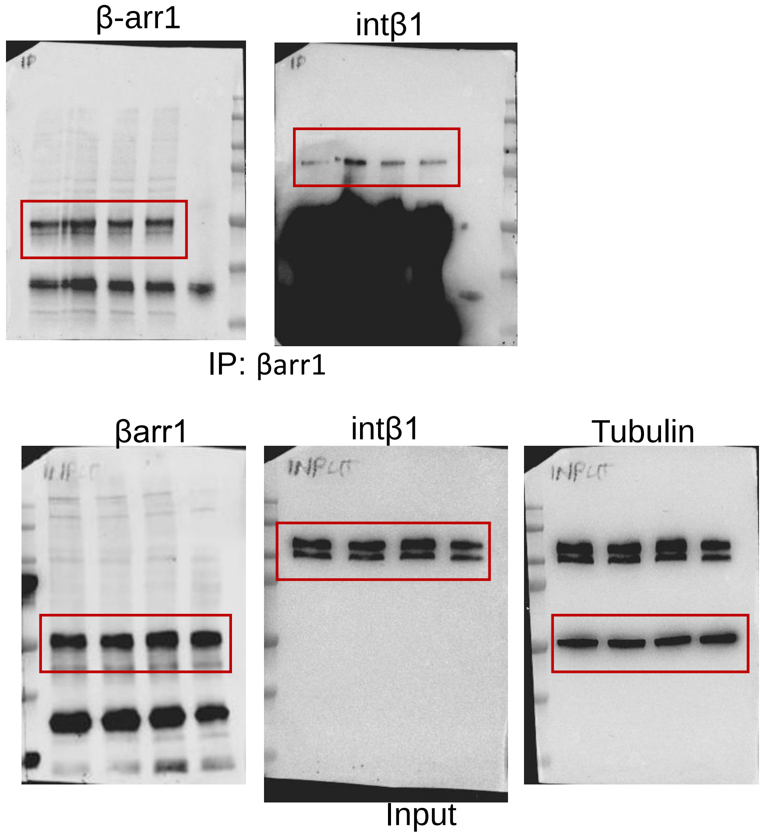

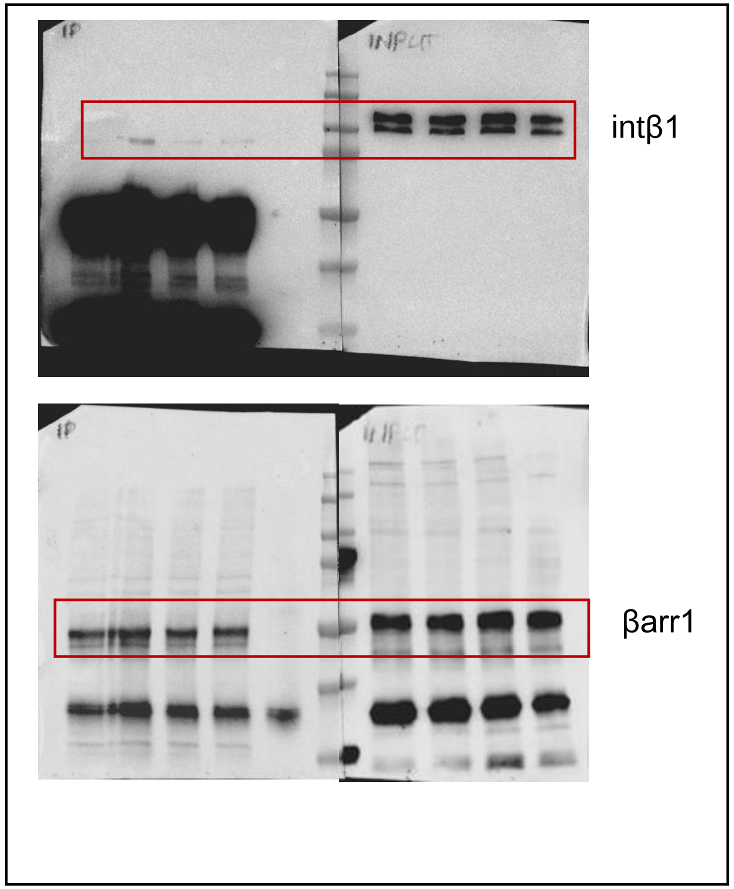

UNCUTTED

Figure 5C

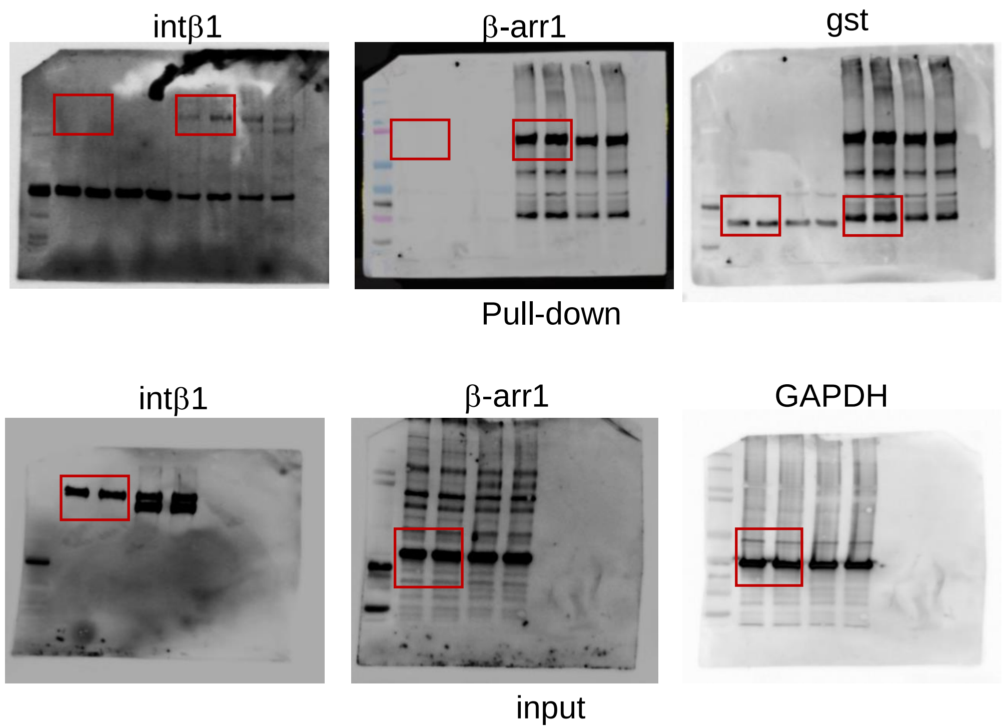

Figure 5D

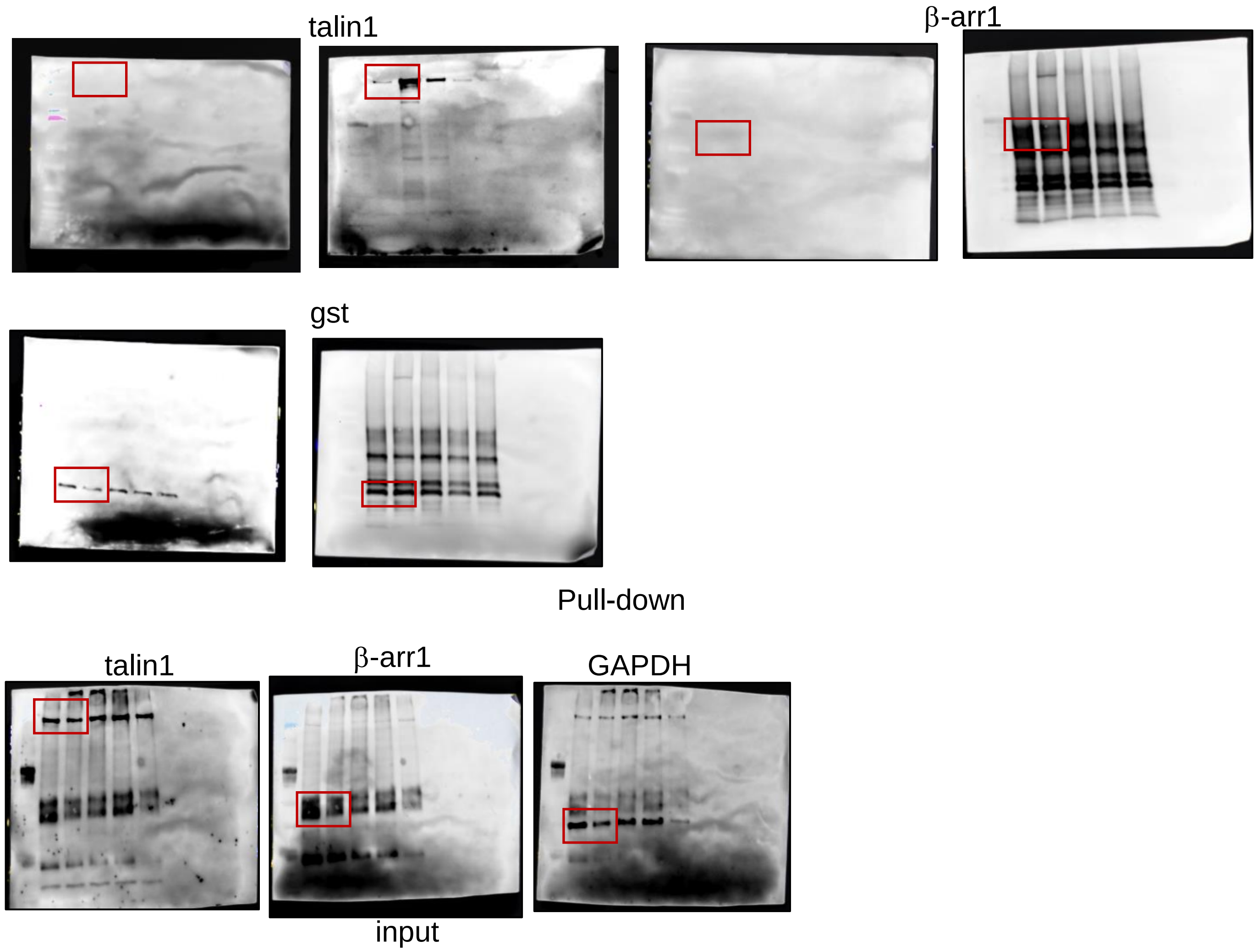

Figure 8C

intβ1

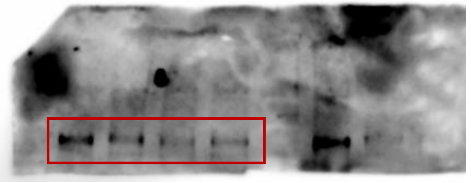

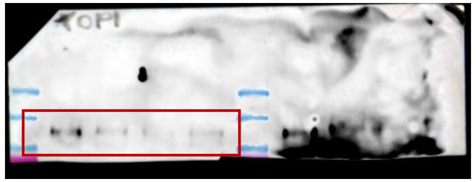

GAPDH

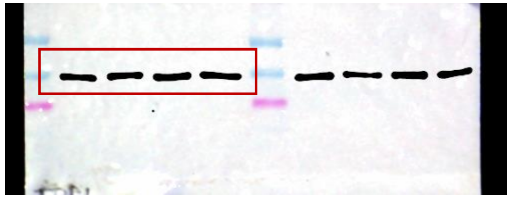

Figure S2A

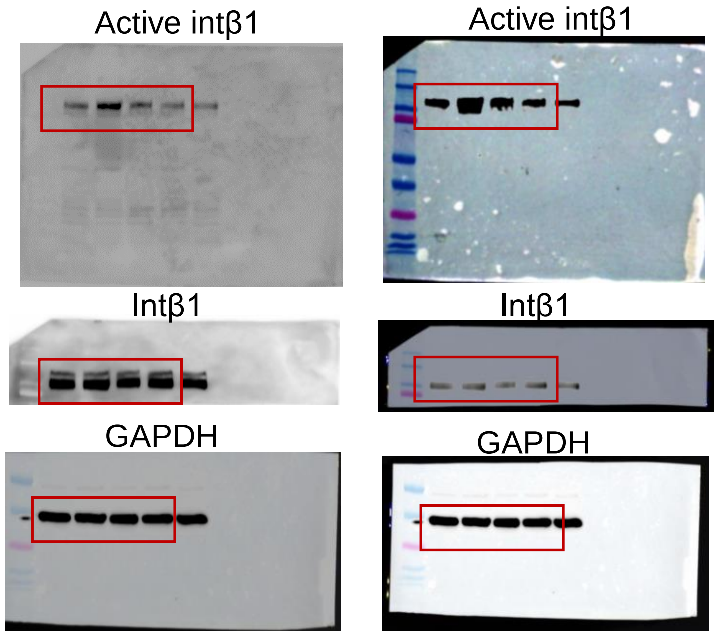

Figure S2B

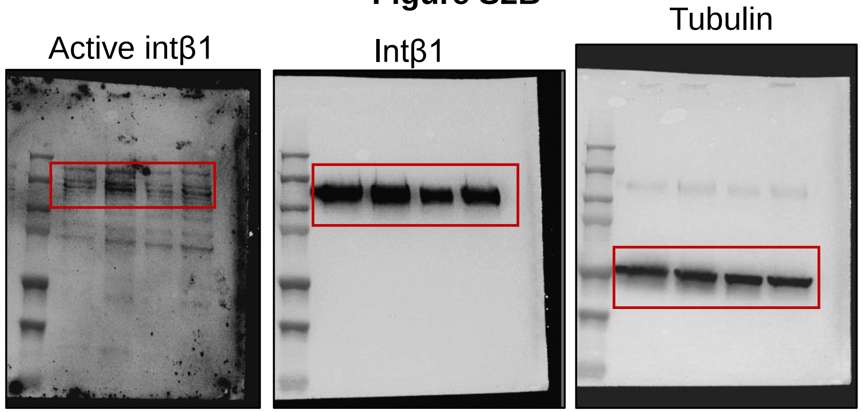

Figure S2C

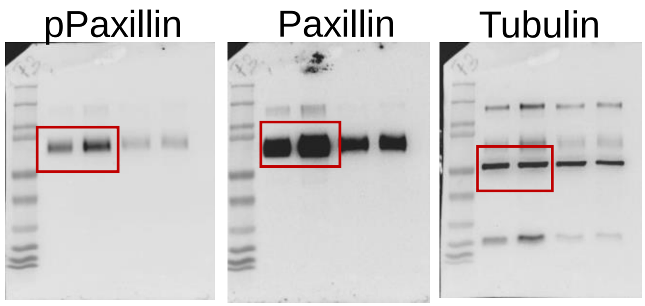

Figure S2D

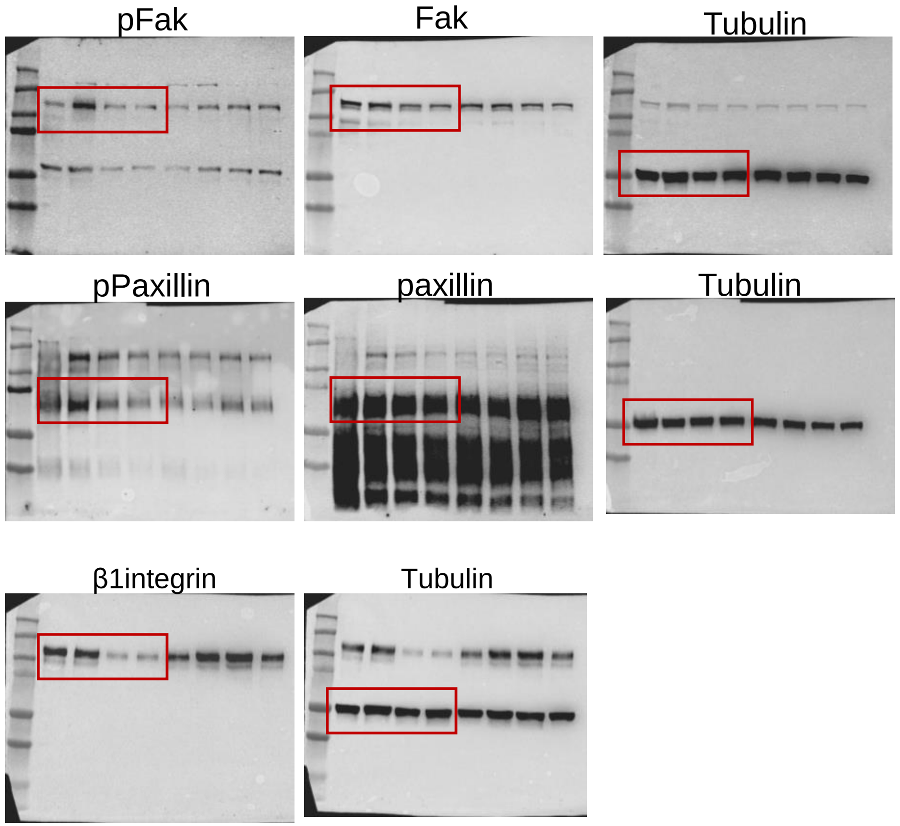

Figure S2E

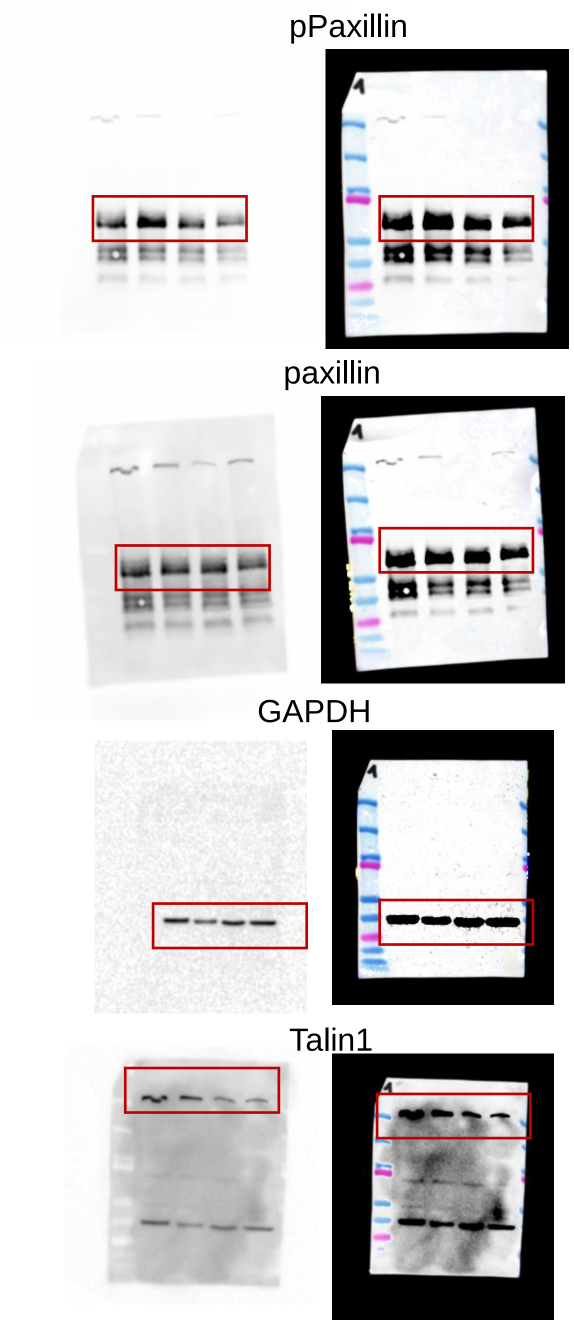

Figure S3A

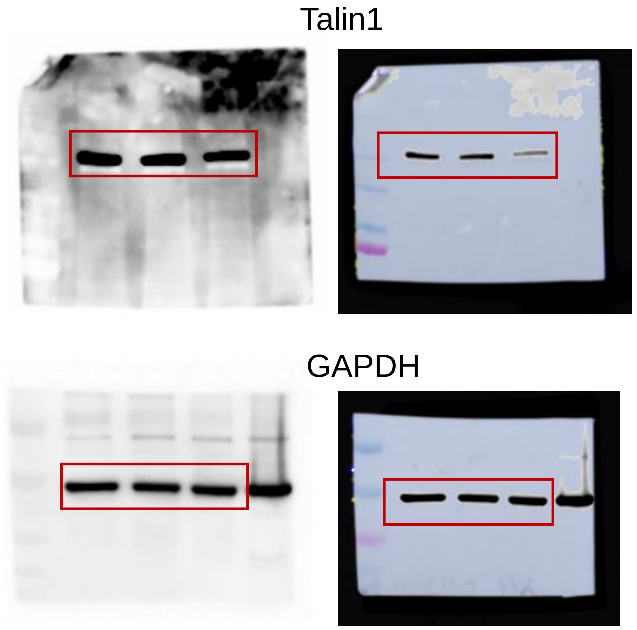

Figure S3B

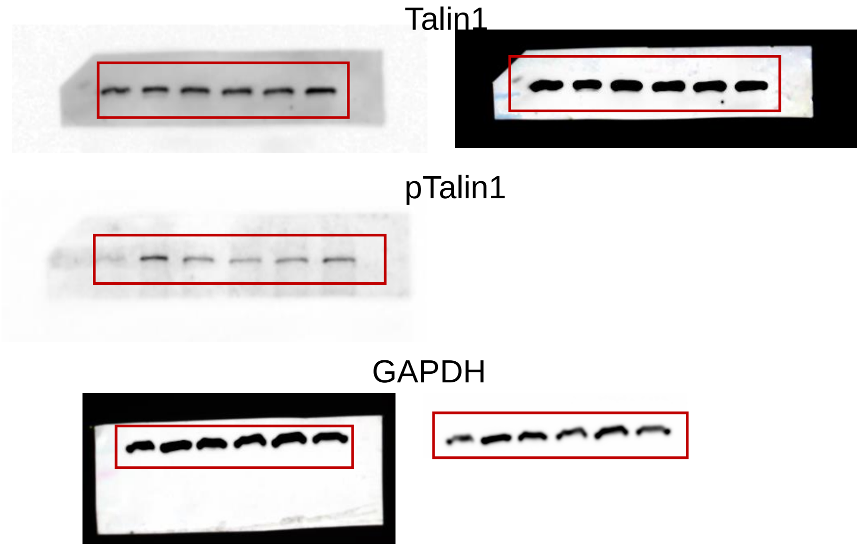

Figure S4B

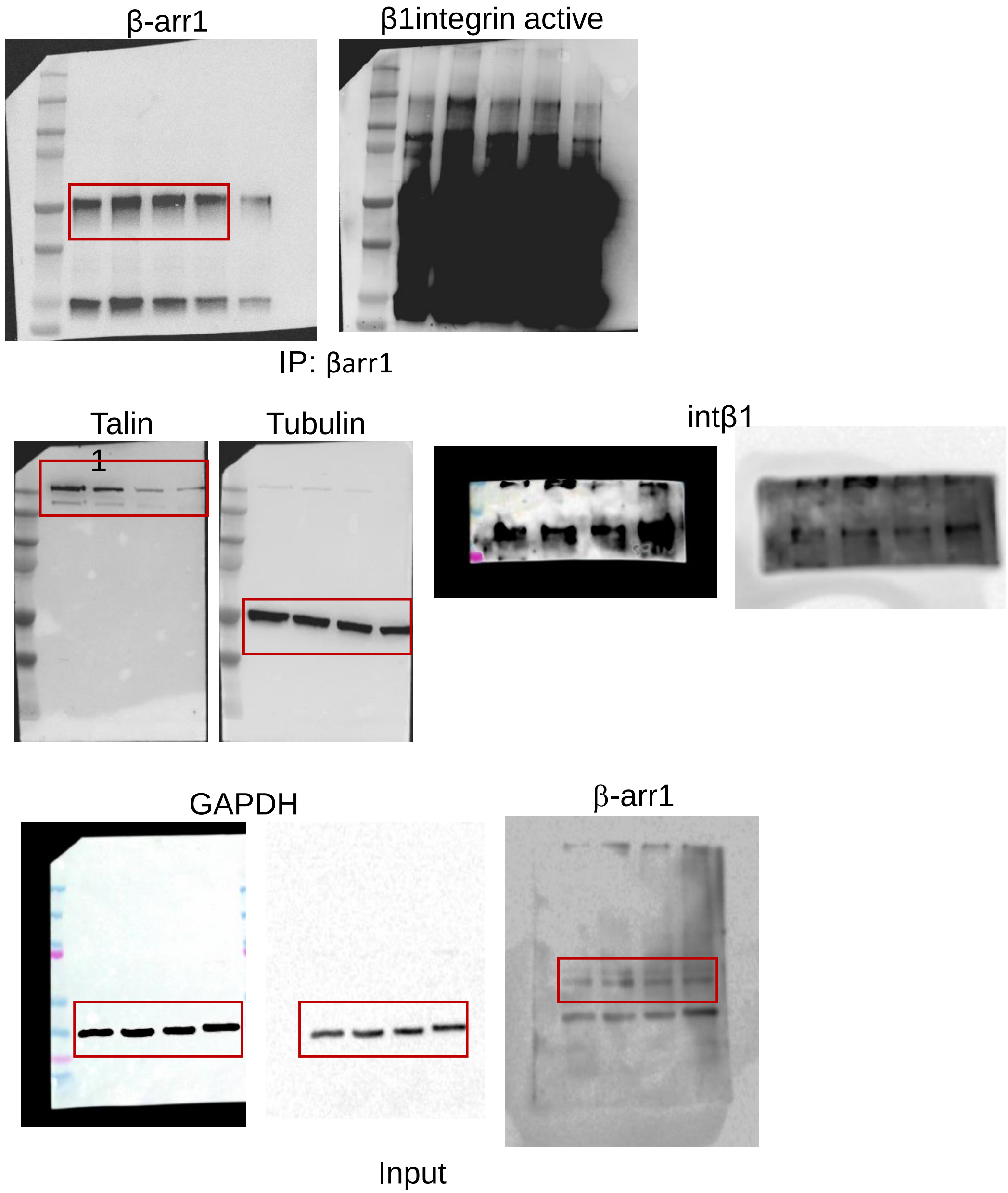

Figure S4C

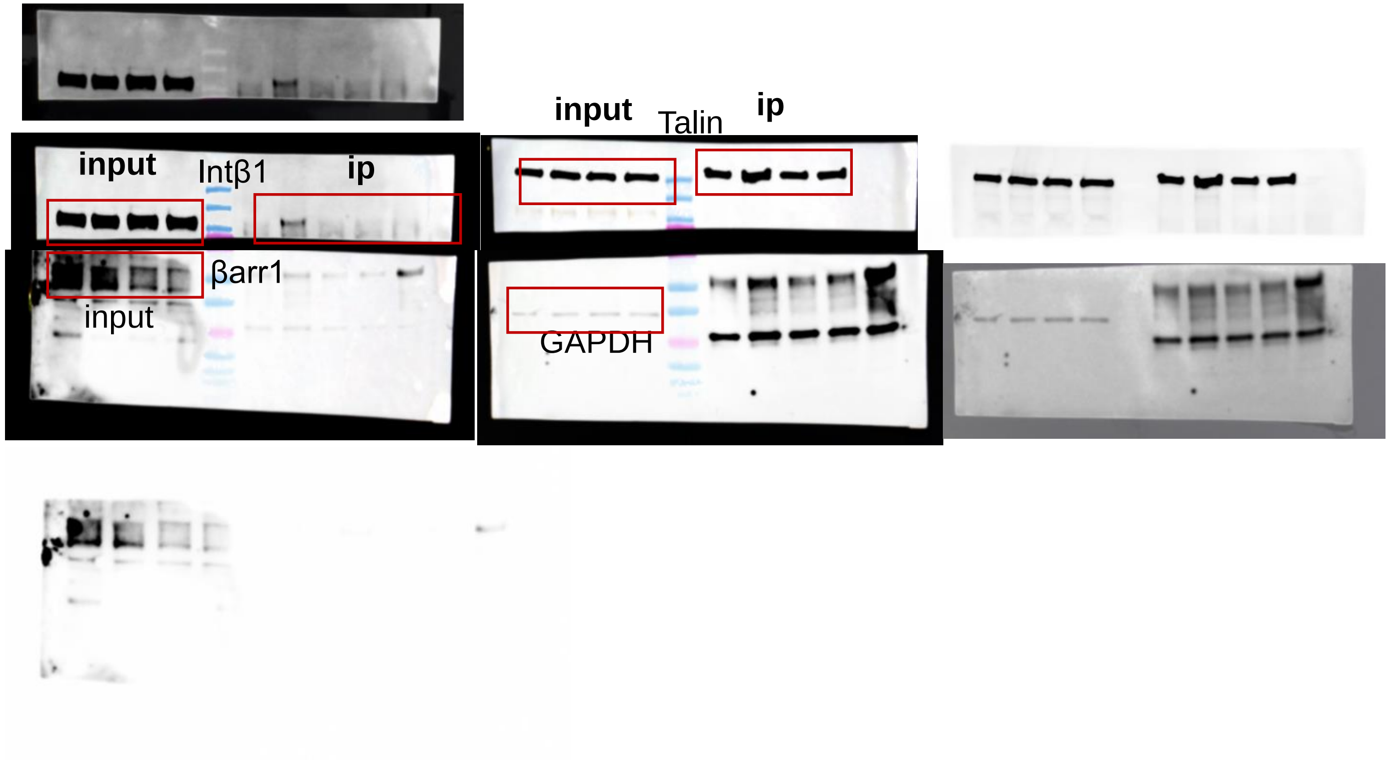

Figure S7B

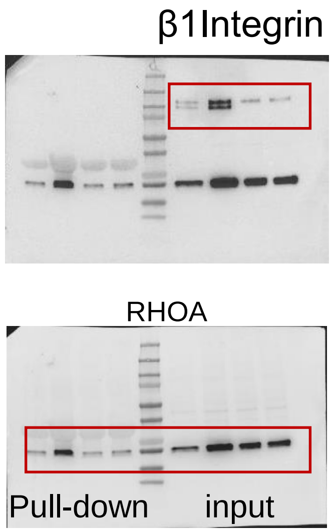

Supplement: Supplementary file 13 — Original Data File [file 41419_2023_5612_MOESM13_ESM.pdf]
